# Supplementary material for: Structural optimization of drug molecules with incrementally trained language models
Source: Nat Commun. 2026 Apr 11;17:3456. doi: 10.1038/s41467-026-71591-w (PMC13076696; doi:10.1038/s41467-026-71591-w)
Supplement: Supplementary file 1 — Supplementary Information [file 41467_2026_71591_MOESM1_ESM.pdf]

Structural optimization of drug molecules with incrementally trained language models

- Supplementary Information -

Tim Hörmann[1], Domenic Mayer[1], Max Lewandowski[1], Andrea Hunklinger[1], Thomas Wein[1], Daniel Merk[1]*

[1] Ludwig-Maximilians-Universität München, Department of Pharmacy, 81377 Munich, Germany

* daniel.merk@cup.lmu.de

Table of Contents

Description of Supplementary Information Items 2
Supplementary Figures 3
Supplementary Tables 7
Synthetic procedures and analytical data 25
Reporter gene assays 38
Supplementary References 39

Description of Supplementary Information Items

pparg_prospective_designs_all_[1-5].xls contain the CLM designs obtained from models fine-tuned with all PPARγ template molecules (repeated five times)
pparg_prospective_designs_all_1_top100.xls contains the top-100 CLM designs obtained from a model fine-tuned with all PPARγ template molecules

pparg_prospective_designs_best_[1-5].xls contain the CLM designs obtained from models fine-tuned with only the most active PPARγ template molecules (repeated five times)
pparg_prospective_designs_best_1_top100.xls contains the top-100 CLM designs obtained from a model fine-tuned with only the most active PPARγ template molecules

pparg_prospective_designs_inc5_[1-5].xls contain the CLM designs obtained from models fine-tuned that were incrementally fine-tuned with increasingly potent PPARγ template molecules (repeated five times)
pparg_prospective_designs_ inc5_1_top100.xls contains the top-100 CLM designs obtained from a model that was incrementally fine-tuned with increasingly potent PPARγ template molecules

pretraining_data.xls contains the data for the pretraining of PPARγ or RORγ naïve CLMs, respectively

retrospective_results_Fig2h_Fig4c.xlsx contains the results of retrospective evaluation summarized in Figures 2h and 4c

rorg_prospective_designs.xlsx contains the CLM designs obtained in the prospective application on RORγ
rorg_prospective_designs_top_10.xlsx contains the top-10 CLM designs obtained in the prospective application on RORγ

sar_series.xlsx contains the PPARγ and RORγ ligand SAR series used for CLM fine-tuning

Supplementary Figures

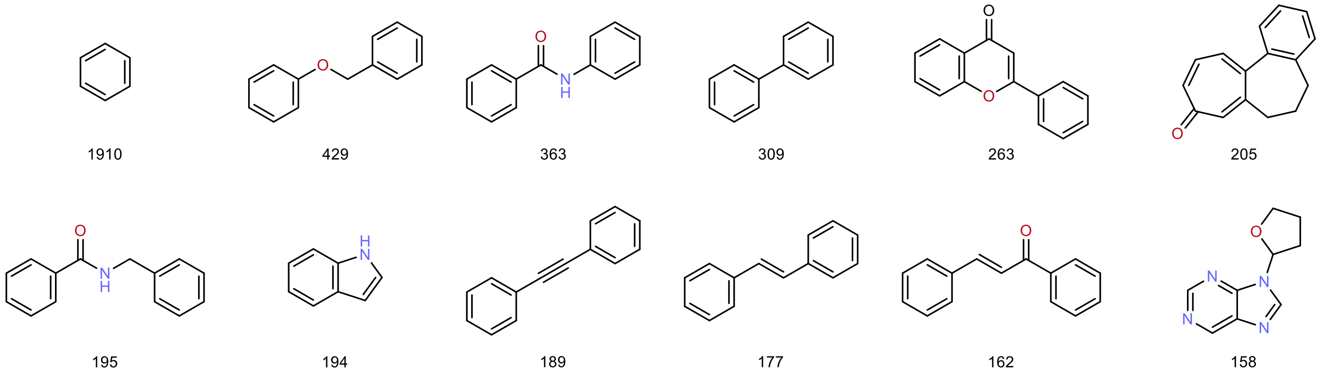

Suppl. Fig. 1. Most frequent scaffolds removed from the pretraining dataset by filtering off PPAR ligands. The numbers refer to the corresponding number of compounds per scaffold. Compounds with no Bemis–Murcko scaffold were removed 596 times.

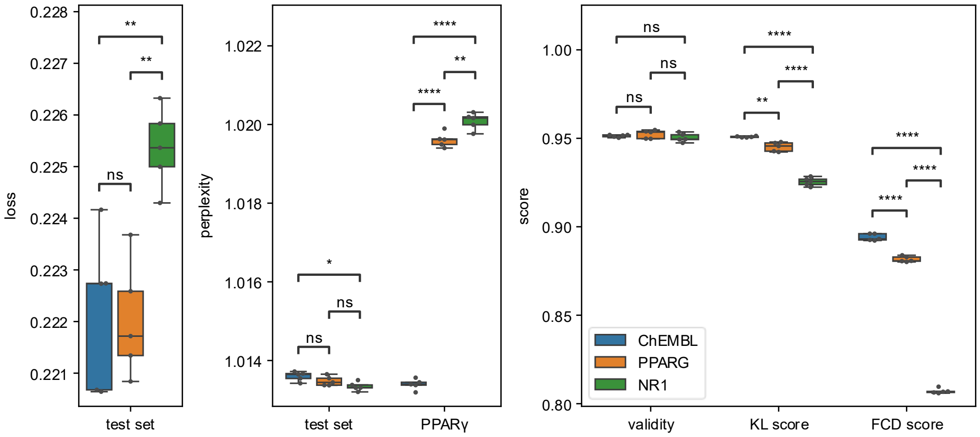

Suppl. Fig. 2. Impact of differently filtered pretraining data (full-ChEMBL, PPAR-ignorant, and NR1-ignorant) on the perplexity of known PPARγ ligands and on CLM performance. The PPAR-ignorant model displayed higher perplexity for known PPARγ ligands than the model trained with the full ChEMBL dataset, while the difference in perplexity between the PPAR-ignorant and the NR1-ignorant model was negligible despite reaching nominal statistical significance. While validity remained consistent across all three strategies, other distribution metrics (KL score and FCD score) were inferior for the NR1-ignorant model relative to the PPAR-ignorant and full-ChEMBL models. Test set loss and perplexity represent the mean over 10,000 random molecules from their respective test sets (n=5 pretraining runs). Perplexity for known PPARγ modulators is reported as the mean over 3,695 PPARγ ligands with no activity cutoff. Validity was determined using 10 k generated SMILES (n=5). Distributional similarity was quantified using the KL Score (scaled Kullback–Leibler divergence across 10 chemical descriptors, n=5) and the Fréchet ChemNet Distance (FCD, also scaled, n=5) calculated between 10 k unique and novel samples and a random subset of 10 k molecules from their respective test sets. Box plots show median and interquartile range (IQR), with whiskers extending to the most extreme points within 1.5 × IQR. Statistical significance was evaluated using ANOVA with Tukey's post-hoc test. Source data are provided as a Source Data file.

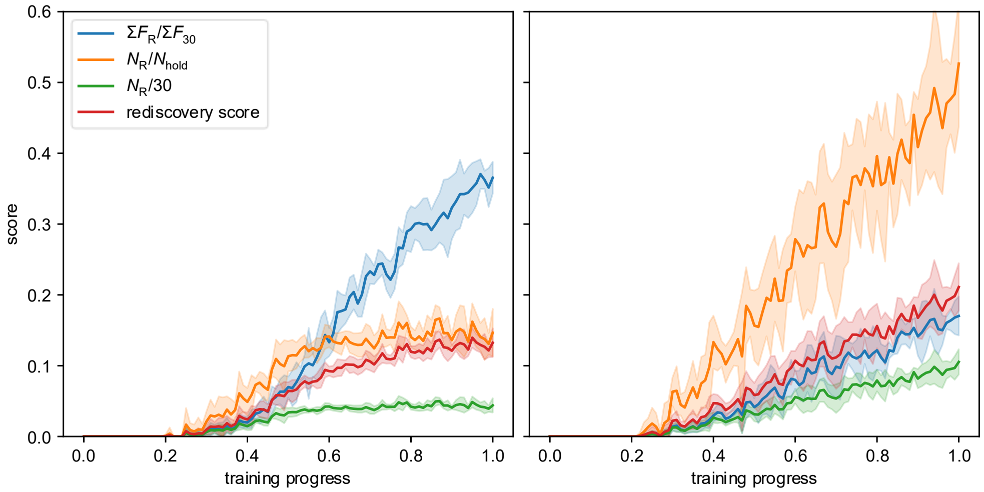

Suppl. Fig. 3. Monitoring of the rediscovery score and its sub-metrics over fine-tuning procedures (*all* method) with two exemplative PPARγ ligand datasets (without the respective holdout sets). Left example: only few holdout molecules are rediscovered (~10%) but sampled disproportionately often (~40% of the top-30 designs). Right example: many holdout molecules are rediscovered (~50%) but sampled rarely (< 20% of the top-30 designs). Although one sub-metric is constantly rising in each example (left: sampling frequency; right: fraction of rediscovered molecules), the rediscovery score increases only moderately and remains at a moderate level demonstrating that the score balances the performance measures and is robust against extreme cases. The training progress is scaled by epochs to 0-1; the score and sub-metrics are shown as mean (lines) with SD (shades); n=5 fine-tuning runs; N_R - number of rediscovered molecules in top-30 (perplexity ranking); N_{hold} - number of molecules in the holdout set; ΣF_{30} - cumulative sample frequency of all top-30 (perplexity ranking) molecules; ΣF_{R} - cumulative sample frequency of rediscovered molecules in top-30 (perplexity ranking). Source data are provided as a Source Data file.

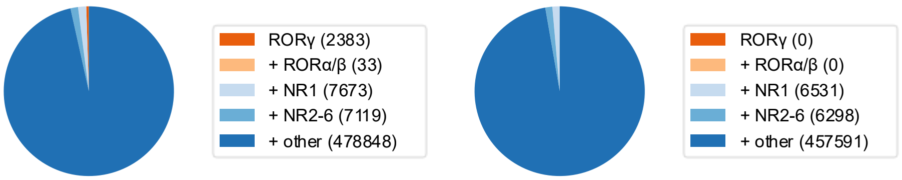

Suppl. Fig. 4. Distribution of molecules in the pretraining data for ROR ignorant CLMs with respect to activity on RORs and related targets before (left) and after (right) the activity- and similarity-based filtering.

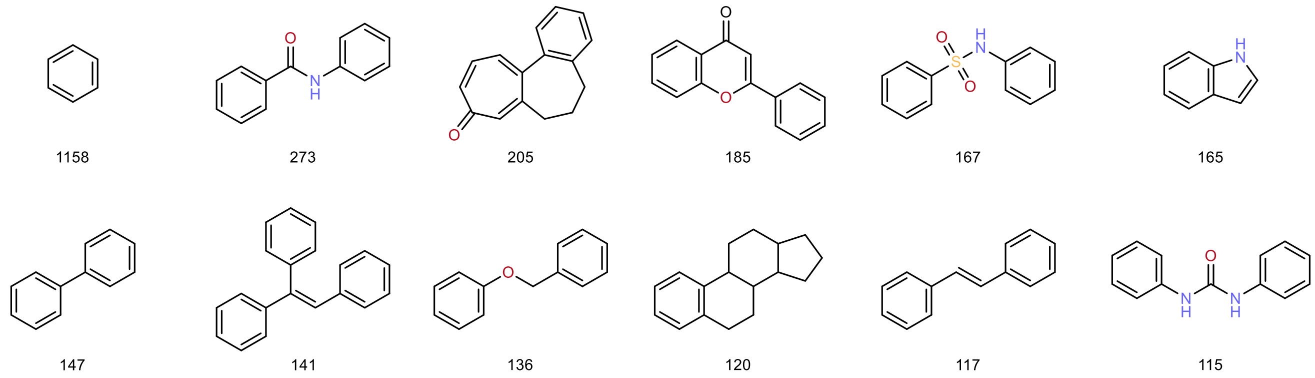

Suppl. Fig. 5. Most frequent scaffolds removed from the pretraining dataset by filtering off ROR ligands. The numbers refer to the corresponding number of compounds per scaffold. Compounds with no Bemis–Murcko scaffold were removed 307 times.

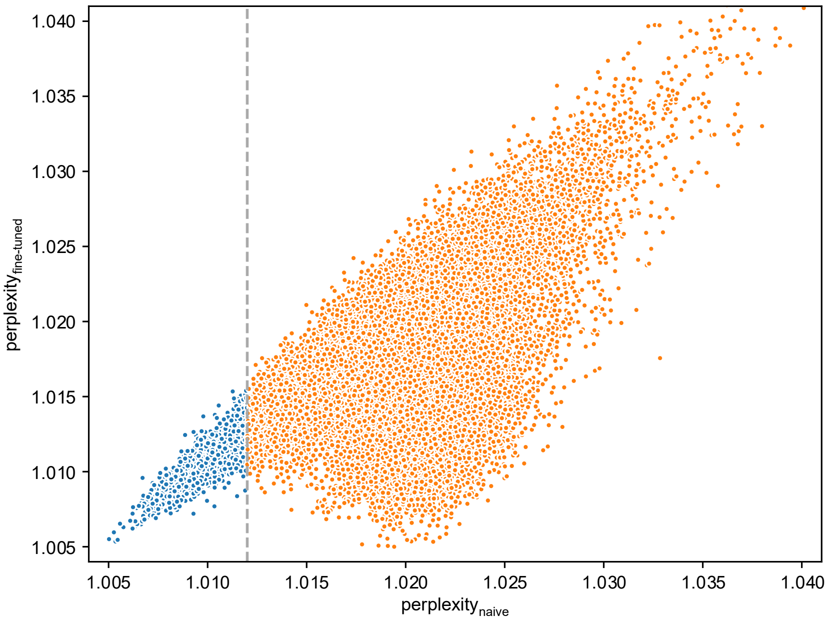

Suppl. Fig. 6. CLM designs with a perplexity < 1.012 (blue region) were excluded as designs sampled by a fine-tuned CLM with very low perplexity also displayed very low perplexity in the naïve model and were thus considered as pre-training artefacts. The figure shows the perplexity of 50 k designs (47k valid, 43.7k unique) from a fine-tuned CLM (PPARγ agonists, *all* method) in the fine-tuned model (y-axis) and the naïve model (x-axis). Source data are provided as a Source Data file.

```
function GetPerplexity(clm, smiles)
    tokens ← Tokenize(smiles)
    probabilities ← clm.Predict(tokens)
    return CalculatePerplexity(tokens, probabilities)

function GetPerplexityScore(clm, smiles)
    if not IsValid(smiles) then
        return GetPerplexity(clm, smiles)
    scores ← [ ]
    for i = 1 to N_variants do
        variant ← Mutate(smiles)
        ppl ← GetPerplexity(clm, variant)
        scores.Push(ppl)
    return Mean(scores)                                          ▷ Average perplexity score

function IncrementalFineTuning(pretrained_weights, data)
    clm ← InitializeCLMWithAdam(pretrained_weights)

    sorted_data ← SortByActivity(data)                           ▷ Splitting
    ft_sets ← SplitIntoEqualSets(data, N_splits)

    for set in ft_sets do                                        ▷ Training
        train, val ← TrainTestSplit(set, train_size = 0.9)
        val_losses ← [ ]
        for i = 1 to N_epochs do
            train_variants ← Mutate(train)
            val_variants ← Mutate(val)
            val_loss ← clm.TrainEpoch(train_variants, val_variants)
            val_losses.Push(val_loss)
        smooth_losses ← SmoothMovingAverage(losses, window = 10)
        clm ← LoadBestEpoch(argmin(smooth_losses))

    generated ← { }
    for i = 1 to N_samples do                                    ▷ Sampling
        smiles ← clm.Generate()
        ppl ← CalculatePerplexityScore(clm, smiles)
        if smiles in generated then
            previous ← generated[smiles]
            previous.ppl ← min(previous.ppl, ppl)
            previous.freq ← previous.freq + 1
        else
            generated[smiles] ← SMILES(smiles, ppl, 1)

    naive_clm ← InitializeCLM(pretrained_weights)
    filtered_smiles ← [ ]
    for smiles_obj in generated do                               ▷ Filtering
        smiles ← smiles_obj.smiles
        naive_ppl ← CalculatePerplexityScore(naive_clm, smiles)
        if
            not IsValid(smiles)                                  ▷ Invalid SMILES
            or smiles in data                                    ▷ Present in training
            or IsKnown(smiles)                                   ▷ Found in ChEMBL/SureChEMBL
            or IsBelowNaiveThreshold(naive_ppl)                  ▷ Pretraining artifact
            or ExceedsSimilarity(smiles, data)                   ▷ Tanimoto > threshold
            or IsSubstructure(smiles, data)
            or IsIsomer(smiles, data)
            or IsInTop100All(smiles)                             ▷ In "all" strategy top results
             then continue
        else
            filtered_smiles.Push(smiles_obj)
    return SortByPerplexity(filtered_smiles)
```

Suppl. Fig 7. Pseudocode for the incremental fine-tuning process.

Supplementary Tables

Suppl. Tab. 1. Molecules used for the fine-tuning of the CLM for prospective PPARγ ligand design sorted by potency. The most potent compound **A** (last entry) is highlighted in blue. Different fine-tuning batches are separated by horizontal lines.

Structure	Fine-tuning batch	Name/ID in original source	PPARγ EC_{50} [μM]	Reference
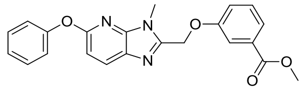	1	5b	12.4	[1]
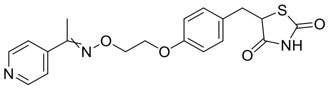	1	4d	7.9	[2,3]
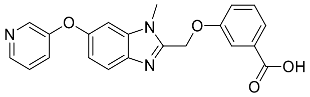	1	1a	6.831	[4]
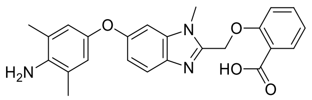	1	8h	6.094	[5]
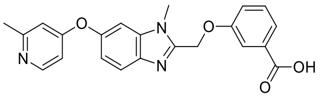	1	2a	5.947	[4]
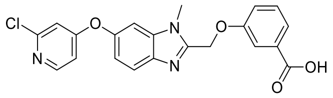	1	2e	5.947	[4]
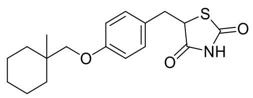	1	Ciglitazone	3.0	[3]
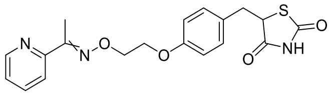	1	4b	2.8	[2,3]
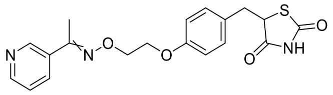	1	4c	2.8	[2,3]
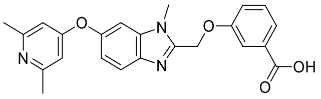	1	2f	2.745	[4]
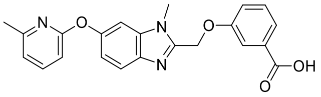	1	16f	1.895	[1]
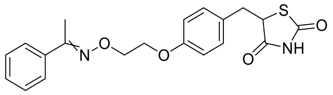	1	4a	1.7	[2,3]
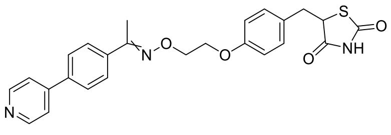	1	4o	1.6	[2,3]
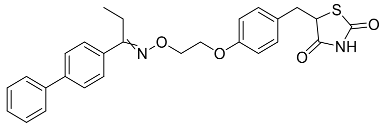	1	4g	1.6	[2,3]
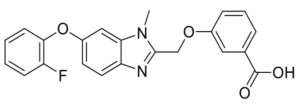	1	13b	1.434	[5]

Structure	Fine-tuning batch	Name/ID in original source	PPARγ EC_{50} [μM]	Reference
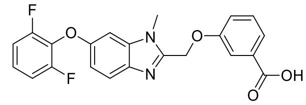	1	13s	1.326	[5]
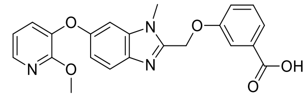	1	1f	1.193	[4]
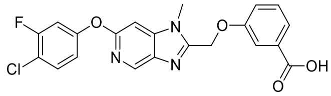	1	6k	1.174	[1]
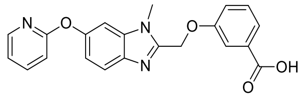	1	16a	1.132	[1]
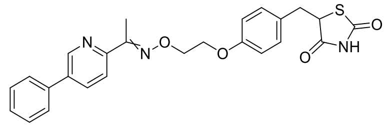	1	4k	1.1	[2,3]
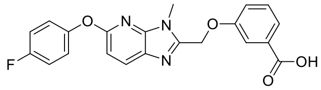	1	6c	1.060	[1]
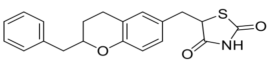	1	Englitazone	1.0	[3]
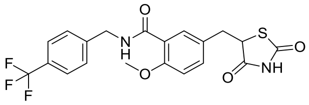	1	KRP297	0.85	[3]
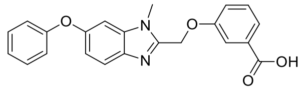	1	13a	0.845	[5]
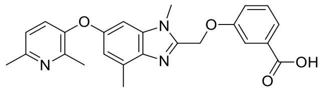	1	3a	0.811	[4]
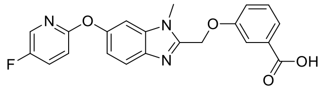	2	16j	0.808	[1]
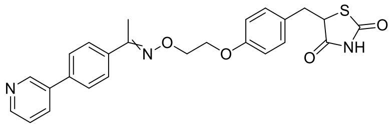	2	4n	0.80	[2,3]
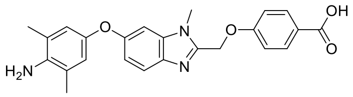	2	8j	0.797	[5]
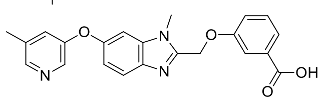	2	1d	0.779	[4]
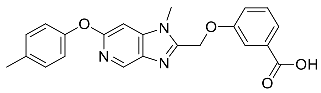	2	6l	0.746	[1]
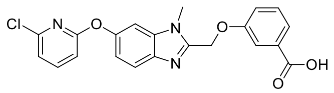	2	16b	0.736	[1]
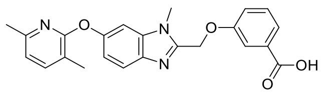	2	16m	0.717	[1]

Structure	Fine-tuning batch	Name/ID in original source	PPARγ EC$_{50}$ [μM]	Reference
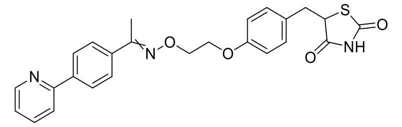	2	4m	0.64	[2,3]
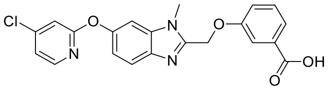	2	16d	0.615	[1]
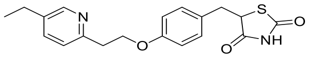	2	Pioglitazone	0.6	[3]
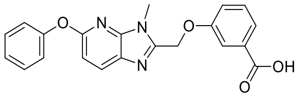	2	6b	0.550	[1]
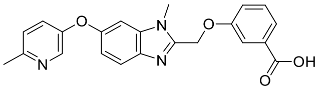	2	1c	0.543	[4]
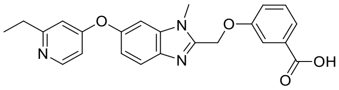	2	2b	0.509	[4]
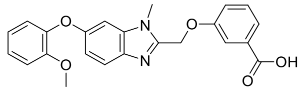	2	13j	0.474	[5]
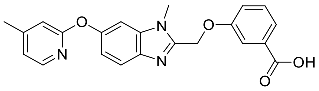	2	16h	0.458	[1]
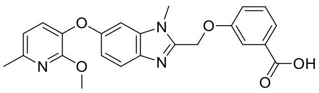	2	1j	0.436	[4]
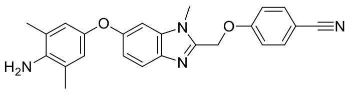	2	8g	0.395	[5]
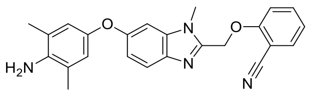	2	8e	0.391	[5]
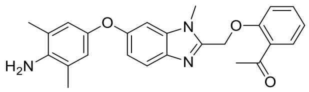	2	8b	0.356	[5]
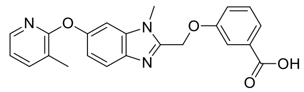	2	16i	0.351	[1]
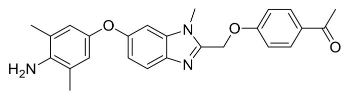	2	8d	0.340	[5]
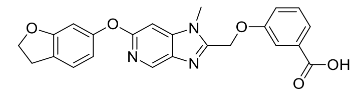	2	6m	0.335	[1]
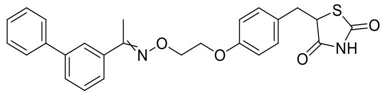	2	4i	0.33	[2,3]
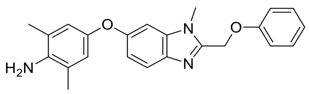	2	8a	0.318	[5]
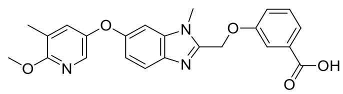	2	1k	0.315	[4]
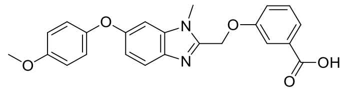	3	13l	0.307	[5]

Structure	Fine-tuning batch	Name/ID in original source	PPARγ EC_{50} [μM]	Reference
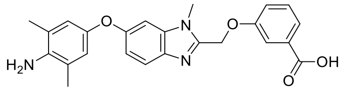	3	8i	0.299	[5]
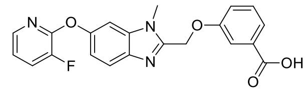	3	16k	0.280	[1]
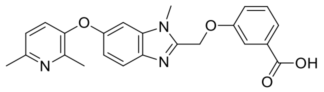	3	1l	0.273	[4]
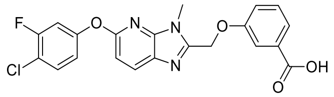	3	6a	0.269	[1]
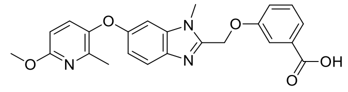	3	1i	0.255	[4]
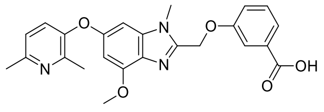	3	3b	0.249	[4]
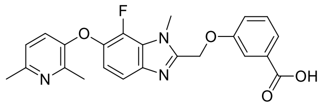	3	3g	0.246	[4]
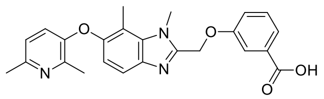	3	3f	0.244	[4]
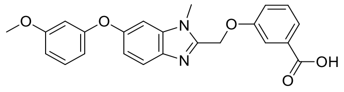	3	13k	0.235	[5]
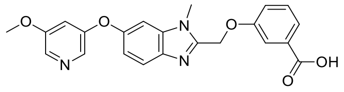	3	1h	0.231	[4]
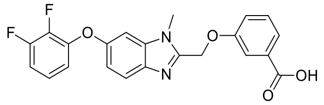	3	13p	0.230	[5]
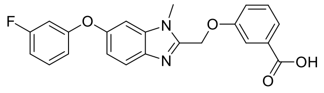	3	13c	0.201	[5]
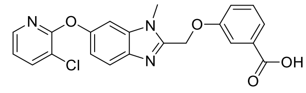	3	16e	0.200	[1]
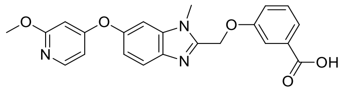	3	2c	0.188	[4]
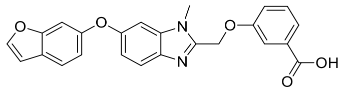	3	13ah	0.188	[5]
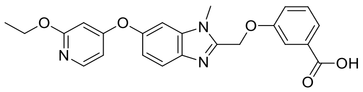	3	2d	0.182	[4]
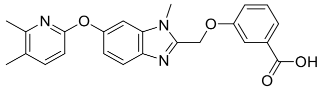	3	16l	0.176	[1]
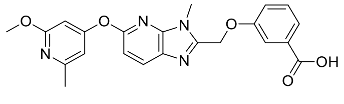	3	2h	0.166	[4]

Structure	Fine-tuning batch	Name/ID in original source	PPARγ EC$_{50}$ [μM]	Reference
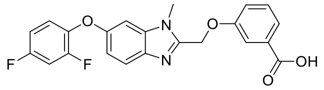	3	13q	0.143	[5]
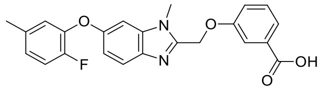	3	13y	0.139	[5]
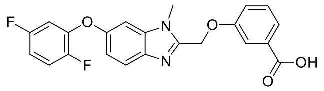	3	13r	0.134	[5]
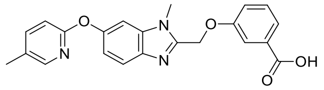	3	16g	0.129	[1]
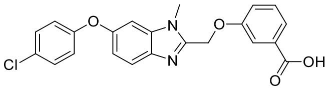	3	13i	0.126	[5]
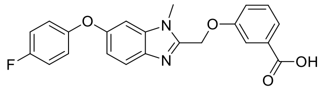	3	13d	0.122	[5]
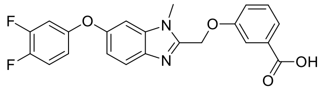	4	13t	0.118	[5]
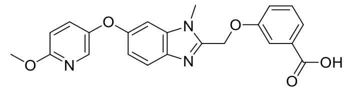	4	1g	0.155	[4]
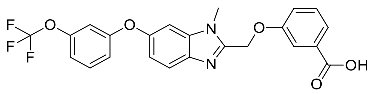	4	13n	0.113	[5]
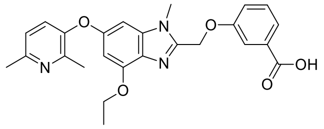	4	3c	0.104	[4]
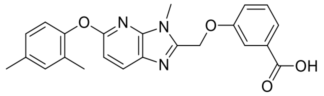	4	6f	0.101	[1]
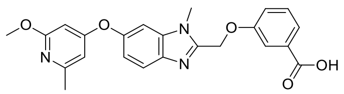	4	2g	0.100	[4]
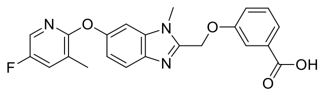	4	16o	0.100	[1]
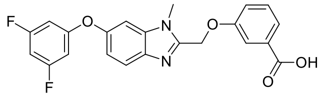	4	13u	0.095	[5]
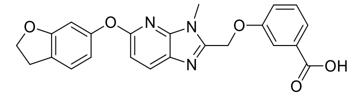	4	6j	0.094	[1]
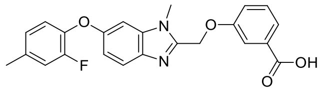	4	13x	0.092	[5]
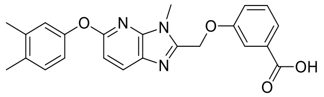	4	6h	0.090	[1]
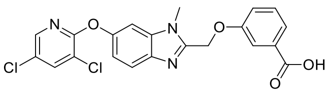	4	16p	0.089	[1]

Structure	Fine-tuning batch	Name/ID in original source	PPARγ EC50 [μM]	Reference
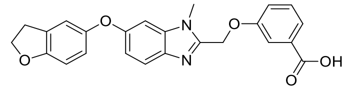	4	13af	0.087	5
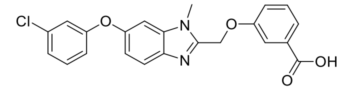	4	13h	0.086	5
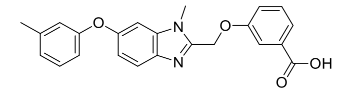	4	13f	0.079	5
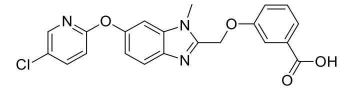	4	16c	0.071	1
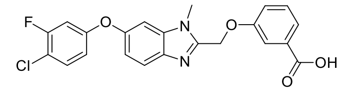	4	13ac	0.068	5
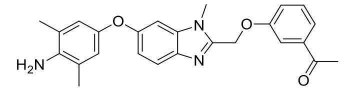	4	8c	0.065	5
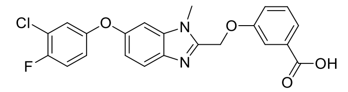	4	13ae	0.064	5
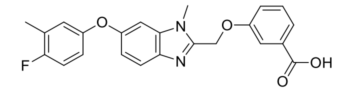	4	13aa	0.062	5
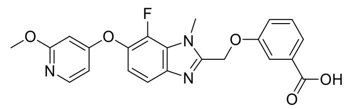	4	5d	0.060	4
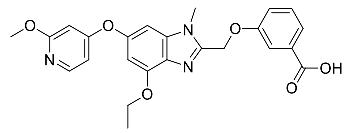	4	5b	0.058	4
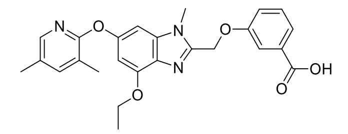	4	4a	0.058	4
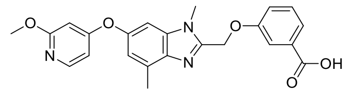	4	5a	0.057	4
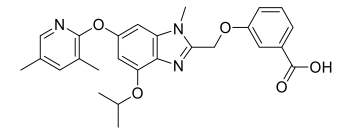	4	4b	0.057	4
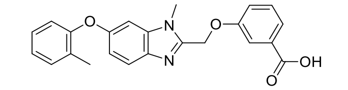	5	13e	0.056	5
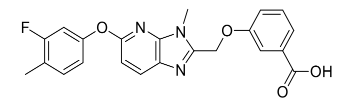	5	6e	0.050	1
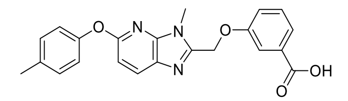	5	6d	0.045	1
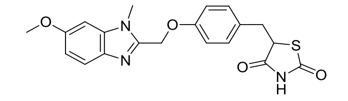	5	II (Rivoglitazone)	0.043	5

Structure	Fine-tuning batch	Name/ID in original source	PPARγ EC_{50} [μM]	Reference
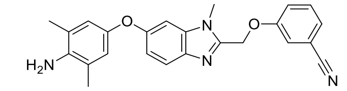	5	8f	0.043	[5]
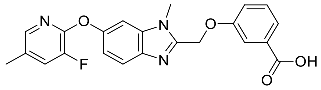	5	16n	0.043	[1]
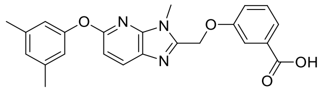	5	6g	0.041	[1]
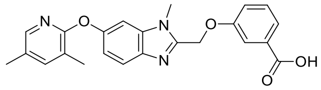	5	I (DS-6930)	0.041	[1]
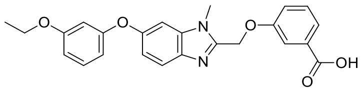	5	13m	0.040	[5]
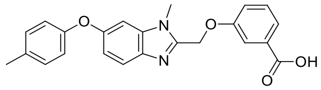	5	13g	0.040	[5]
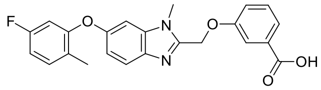	5	13w	0.039	[5]
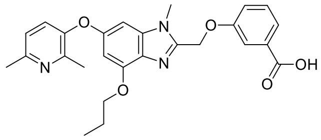	5	3d	0.032	[4]
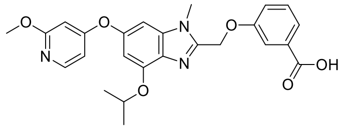	5	5c	0.032	[4]
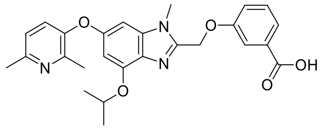	5	3e	0.031	[4]
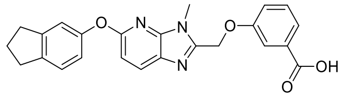	5	6i	0.025	[1]
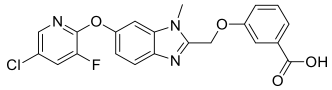	5	16q	0.024	[1]
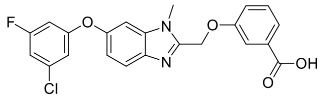	5	13ad	0.023	[5]
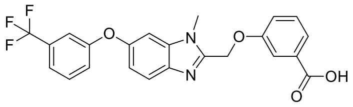	5	13o	0.023	[5]
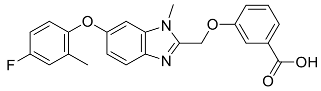	5	13v	0.022	[5]
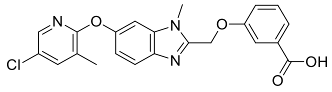	5	16r	0.021	[1]
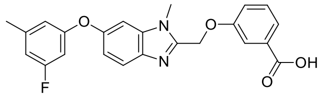	5	13ab	0.021	[5]

Structure	Fine-tuning batch	Name/ID in original source	PPARγ EC$_{50}$ [μM]	Reference
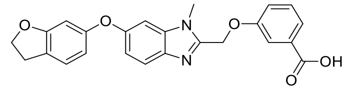	5	13ag	0.021	[5]
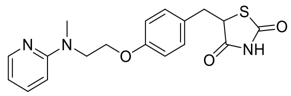	5	Rosiglitazone	0.02	[3]
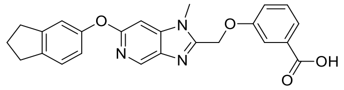	5	6n	0.020	[1]
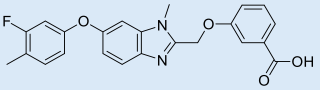 **A**	5	13z	0.0044	[5]

Suppl. Tab. 2. Designs **1-10** from the prospective application to PPARγ and their most similar fine-tuning molecules (cf. Fig. 3f) based on Tanimoto similarity computed on Morgan Fingerprints (radius = 3, 2048 bits). EC_{50} values for the designs were determined in a Gal4- PPARγ hybrid reporter gene assay (n.d. - not determined). EC_{50} values for the most similar fine-tuning molecules from the respective original source (reference).

Design (**ID**)	PPARγ EC_{50} (μM)	Most similar fine-tuning molecule	Similarity	PPARγ EC_{50} (μM)	Reference
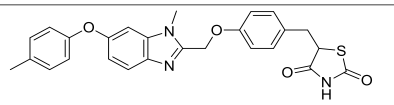 **1**	0.017	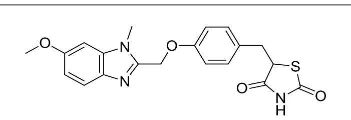	0.75	0.043	[5]
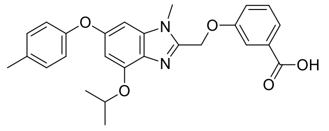 **2**	0.0006	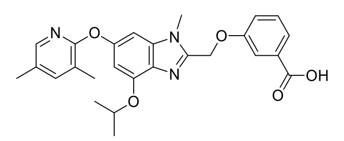	0.74	0.057	[4]
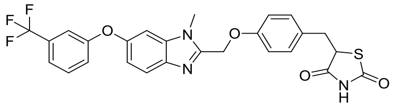 **3**	0.044	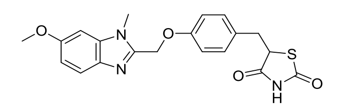	0.67	0.043	[5]
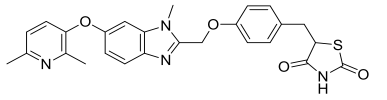 **4**	n.d.	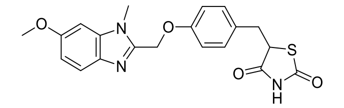	0.71	0.043	[5]
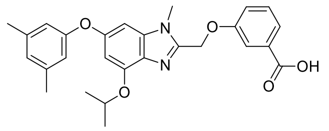 **5**	0.0007	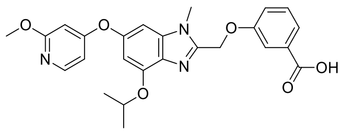	0.73	0.032	[4]
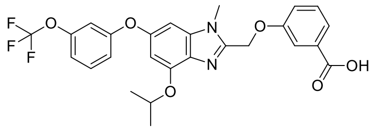 **6**	0.0031	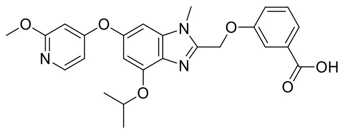	0.69	0.032	[4]
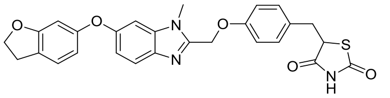 **7**	0.018	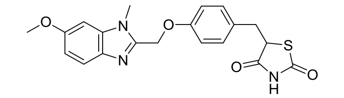	0.64	0.043	[5]
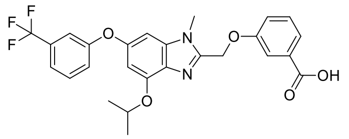 **8**	0.0008	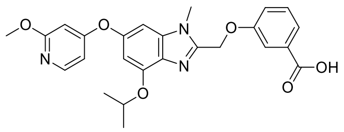	0.70	0.032	[4]
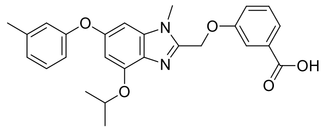 **9**	0.0010	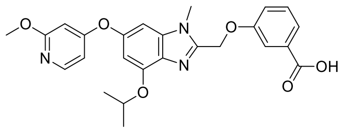	0.75	0.032	[4]
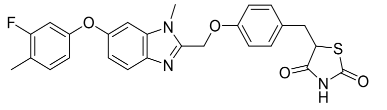 **10**	0.034	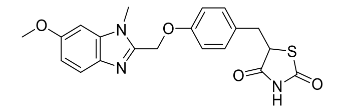	0.71	0.043	[5]

Suppl. Tab. 3. Molecules used for the fine-tuning of the CLM for prospective RORγ ligand design sorted by potency. The reference compounds **B**, **C**, **D**, and **E** are highlighted in blue. The most potent compound of the series of **B**[6] is highlighted in gray. XC_{50} refers to either IC_{50} or EC_{50}, as reported in the original source. Potency data are from reporter gene assays or SRC recruitment assays as reported in the original references. Different fine-tuning batches are separated by horizontal lines.

Structure	Fine-tuning batch	Name/ID in original source	RORγ XC_{50} [µM]	Reference
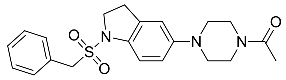	1	4	46.44	6
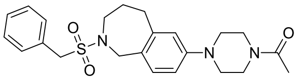	1	10	46.05	6
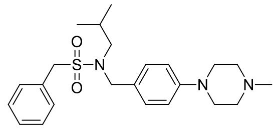	1	8	ChEMBL: 23 µM (2.3)	7
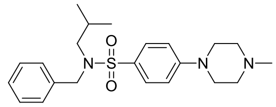	1	15	>20	8
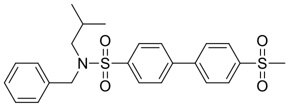	1	12	>20	8
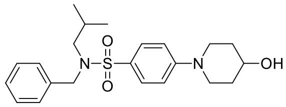	1	14	>20	8
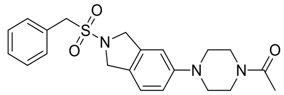	1	3	14.04	6
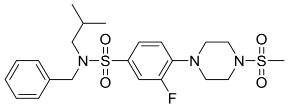	1	21	>10	8
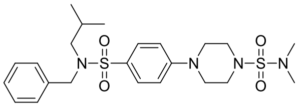	1	17	>10	8
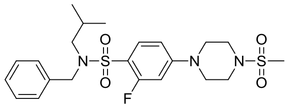	1	20	>10	8
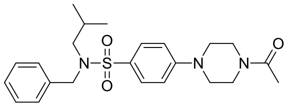	1	16	>10	8
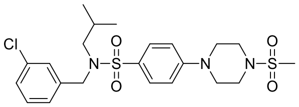	1	29	>10	8
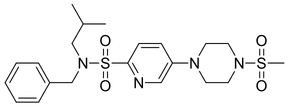	1	24	>10	8
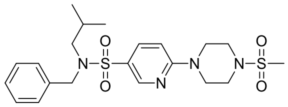	1	26	>10	8
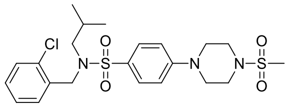	1	28	>10	8

Structure	Fine-tuning batch	Name/ID in original source	RORγ XC_{50} [μM]	Reference
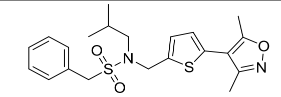	1	18	7.5	9
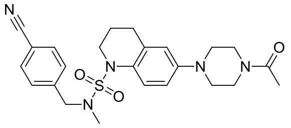	1	33	7.461	6
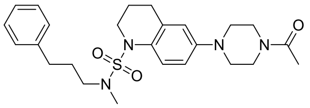	1	15	7.226	6
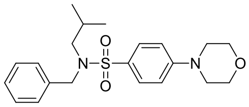	1	13	>6.7	8
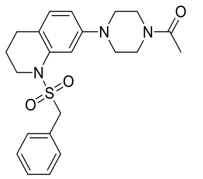	1	7	6.669	6
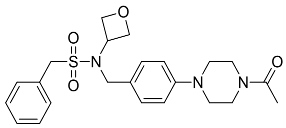	1	19	4.9	7
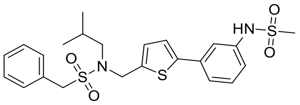	1	9	4.9	9
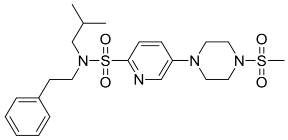	1	25	4.5	8
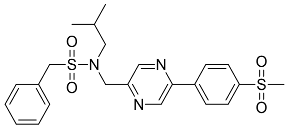	1	24	3.8	9
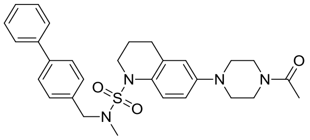	1	31	2.734	6
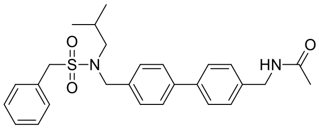	1	28	4.5	9
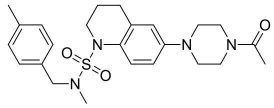	1	23	2.435	6
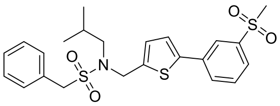	1	2	2.7	9
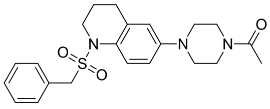	1	8	2.048	6

Structure	Fine-tuning batch	Name/ID in original source	RORγ XC_{50} [μM]	Reference
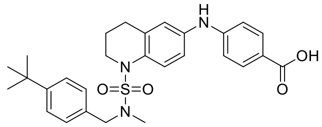	1	38	2.006	[6]
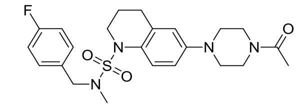	1	20	1.65	[6]
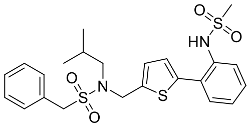	1	8	1.6	[9]
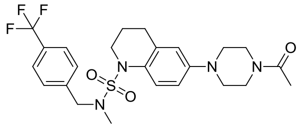	1	26	1.555	[6]
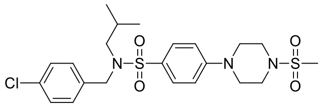	1	30	1.3	[8]
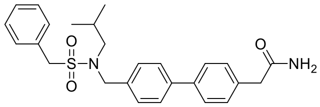	1	29	1.2	[9]
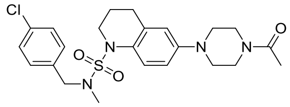	2	29	1.135	[6]
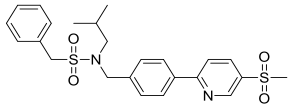	2	23	1.1	[9]
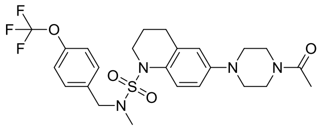	2	32	1.034	[6]
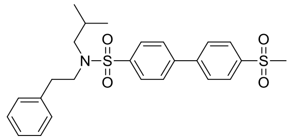	2	10	1.0	[8]
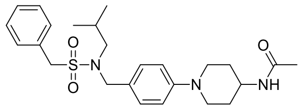	2	16	0.92	[7]
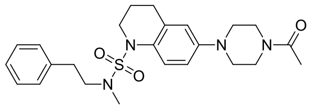	2	14	0.91	[6]
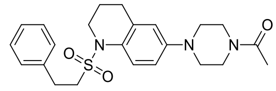	2	12	0.906	[6]
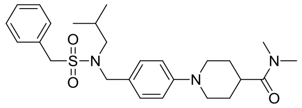	2	13	0.86	[7]
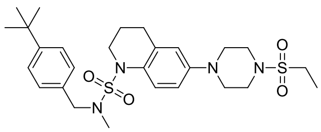	2	35	0.802	[6]

Structure	Fine-tuning batch	Name/ID in original source	RORγ XC_{50} [μM]	Reference
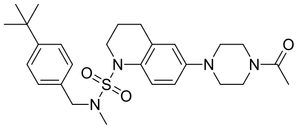	2	30	0.731	[6]
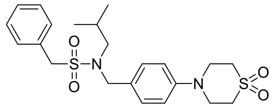	2	6	0.70	[7]
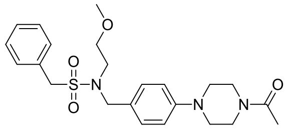	2	25	0.68	[7]
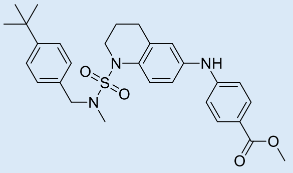 **B**	2	37	0.637	[6]
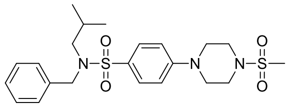	2	18	0.53	[8]
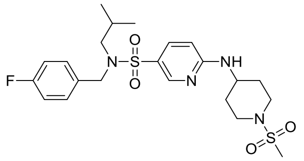	2	48	0.52	[8]
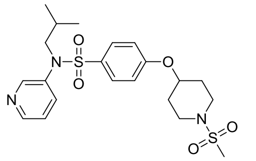	2	46	0.49	[8]
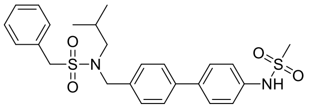	2	25	0.47	[9]
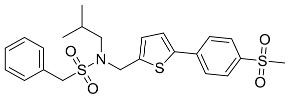	2	7	0.46	[8]
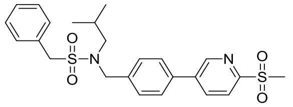	2	22	0.36	[9]
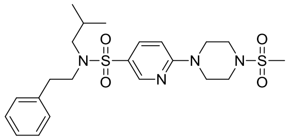	2	27	0.34	[8]
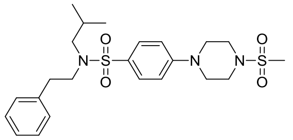	2	19	0.34	[8]
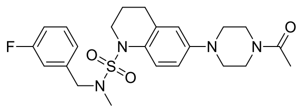	2	19	0.324	[6]

Structure	Fine-tuning batch	Name/ID in original source	RORγ XC_{50} [μM]	Reference
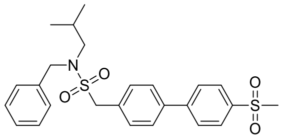	2	11	0.32	8
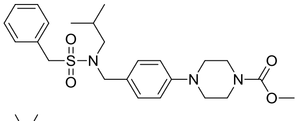	2	15	0.31	7
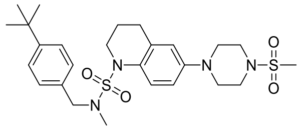	2	34	0.300	6
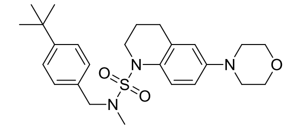	2	36	0.284	6
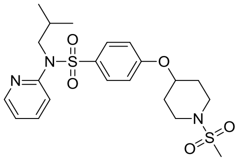	2	45	0.24	8
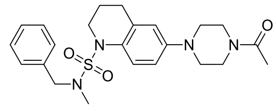	2	13	0.218	6
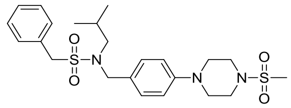	2	3	0.21	7
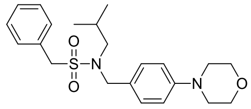	2	7	0.21	7
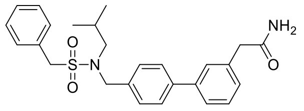	2	30	0.20	9
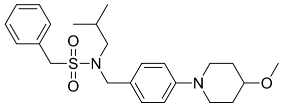	2	10	0.16	7
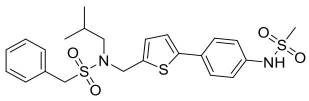	2	10	0.15	9
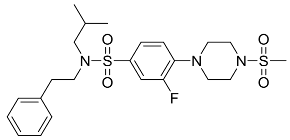	2	23	0.15	8
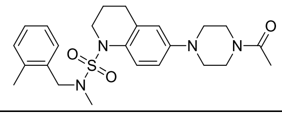	2	21	0.143	6
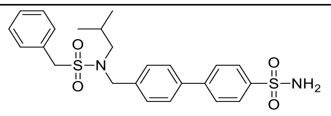	3	26	0.14	9
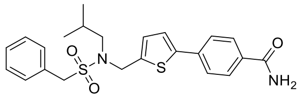	3	11	0.13	9

Structure	Fine-tuning batch	Name/ID in original source	RORγ XC_{50} [μM]	Reference
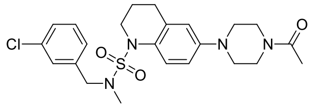	3	28	0.104	[6]
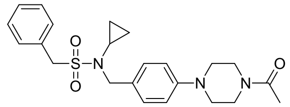	3	22	0.10	[7]
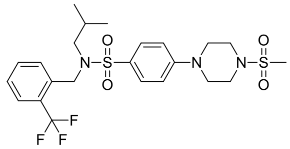	3	31	0.10	[8]
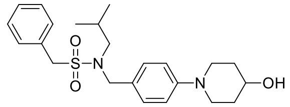	3	9	0.10	[7]
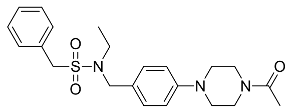	3	23	0.094	[7]
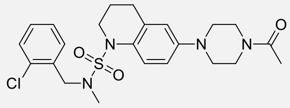	3	27	0.086	[6]
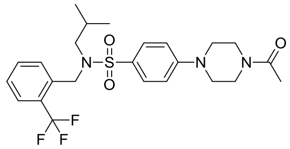	3	32	0.085	[8]
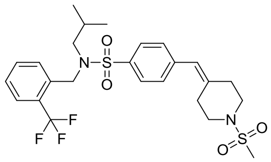	3	38	0.075	[8]
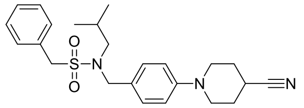	3	11	0.074	[7]
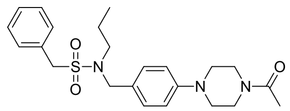	3	20	0.070	[7]
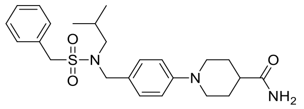	3	12	0.070	[7]
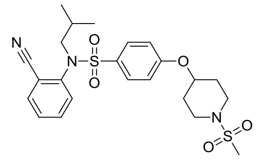	3	42	0.068	[8]
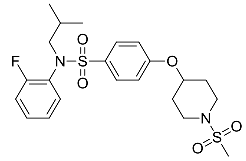	3	39	0.06	[8]

Structure	Fine-tuning batch	Name/ID in original source	RORγ XC_{50} [μM]	Reference
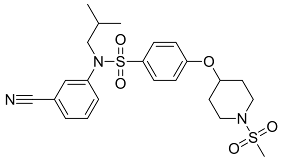	3	43	0.059	8
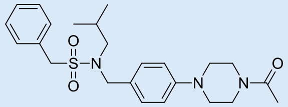 **E**	3	14	0.057	7
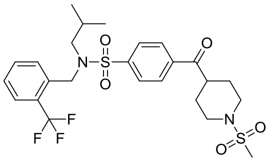	3	36	0.056	8
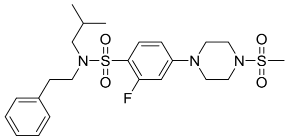	3	22	0.05	8
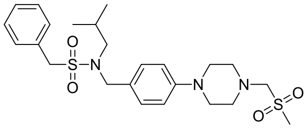	3	5	0.042	7
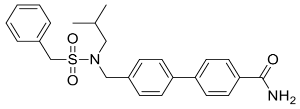	3	8	0.040	8
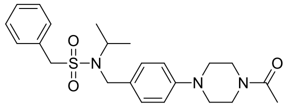	3	21	0.037	7
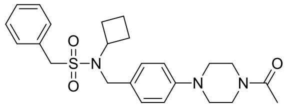	3	17	0.030	7
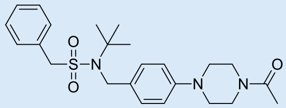 **D**	3	18	0.029	7
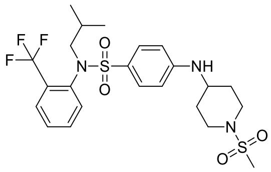	3	34	0.028	8
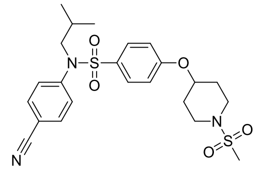	3	44	0.027	8
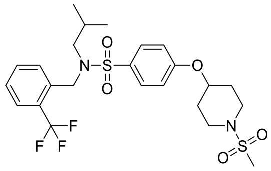	3	35	0.022	8

Structure	Fine-tuning batch	Name/ID in original source	RORγ XC_{50} [μM]	Reference
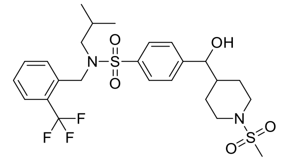	3	37	0.022	[8]
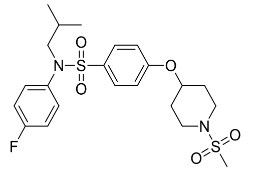	3	41	0.022	[8]
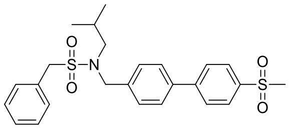	3	9	0.021	[8]
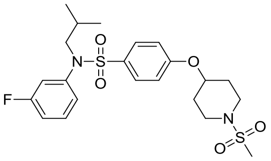	3	40	0.021	[8]
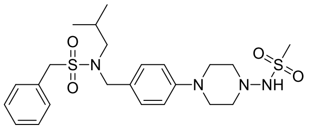	3	4	0.020	[7]
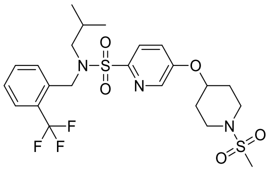	3	47	0.015	[8]
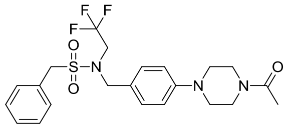	3	24	0.013	[7]
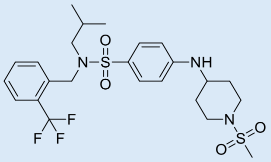 **C**	3	33	0.012	[8]

Suppl. Tab. 4. Designs **27-29** from the prospective application to RORγ and their most similar fine-tuning molecules based on Tanimoto similarity computed on Morgan Fingerprints with feature invariants (radius=2, 512-bit) and on standard Morgan Fingerprints (radius=3, 2048-bit; in parantheses). IC_{50} values for the designs were determined in a Gal4-RORγ hybrid reporter gene assay. XC_{50} values for the most similar fine-tuning molecules from the respective original source (reference).

Design (**ID**)	RORγ IC_{50} (µM)	Most similar fine-tuning molecule	Similarity	RORγ XC_{50} (µM)	Reference
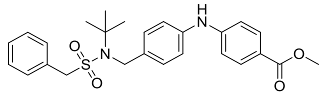 **27**	0.03	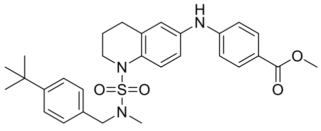	0.57 (0.35)	0.637	6
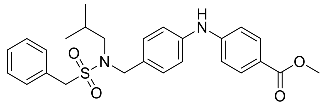 **28**	1.9	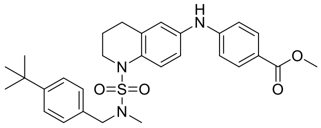	0.59 (0.31)	0.637	6
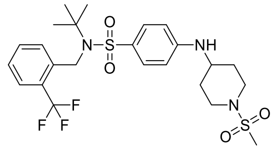 **29**	0.013	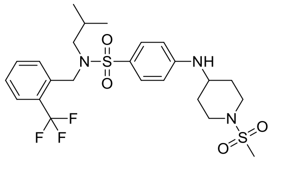	0.85 (0.69)	0.043	8

Synthetic procedures and analytical data

General. All chemicals and solvents were of reagent grade, purchased from commercial sources (e.g., Sigma-Aldrich, abcr, Enamine and BLDpharm) and used without further purification unless otherwise specified. All reactions were conducted in common glassware under air unless otherwise specified. Solvents for synthesis and work-up procedures (EtOAc, EtOH, cyclohexane, ACN), were of reagent grade or purified by distillation. Reactions were monitored by thin layer chromatography (TLC) using fluorescent dye-coated silica gel 60 on aluminum sheets under UV light (254 nm). Purification was performed by automated flash column chromatography (aFCC) on an Interchim puriFlash® XS520Plus on pre-packed silica cartridges (PF-15SIHP-F0025, PF-50SIHP-F0012, PF-50SIHP-F0025 or PF-50SIHP-F0040) with dry loading of the crude product on celite. Reverse phase aFCC was performed on a puriFlash® XS520Plus (Advion) using C18HP Columns (PF-15C18HP-F0012 or PF-15C18HP-F0025). ^{1}H and ^{13}C-NMR spectra were recorded at 25 °C on Bruker Avance III HD 400, or Avance III HD 500 spectrometers (Bruker Corporation, Billerica, MA, USA). Chemical shifts (δ) are reported in parts per million (ppm), coupling constants (*J*) in Hertz (Hz). The NMR spectra were calibrated using the proton or carbon signals of residual non-deuterated solvent peaks (7.26 and 77.00 ppm for $CDCl_3$; 2.05 and 29.84 as well as 206.26 ppm for acetone-d_6; 3.31 and 49.00 ppm for CD_3OD; 2.50, and 39.52 ppm for DMSO-d_6; 5.32 and 53.84 ppm for CD_2Cl_2). Signal multiplicity is reported as follows: s, singlet; brs, broad singlet; d, doublet; dd, doublet of doublets; ddd, doublet of doublets of doublets; dt, doublet of triplets; ddt, doublet of doublets of triplets; dq, doublet of quartets; ddq, doublet of doublets of quartets; t, triplet; td, triplet of doublets; q, quartet; p, pentet; hept, heptet; m, multiplet. For qNMR experiments[10], spectra were acquired at room temperature with 64 scans in a spectral range from -7.5 to 22.5 ppm relative to TMS. Pulse delay was set to 60 s, the acquisition time was 4 s (digital resolution = 0.05 Hz/point) and 2 dummy scans were performed before the acquisition. Low resolution MS spectra were recorded on an Advion expression™ CMS (Advion, Inc., Ithaca, NY, USA) using atmospheric pressure chemical ionization (APCI). High resolution MS analyses were performed on a Finnigan MAT 95 spectrometer (Thermo Fisher Scientific) using electrospray ionization (ESI). All final compounds for biological evaluation had a purity of >95% according to qHNMR.

General synthetic procedures

GP A - Amide Coupling and Benzimidazole Formation: The respective acetic acid derivative (**17**/**19**; 1.0 eq.) and DIPEA (2.0 eq.) were dissolved in DMF (0.1 M) and stirred for 10 min at rt. HATU (2.0 eq.) was added, and the mixture was stirred for another 10 min. Subsequently, the respective amine (**14a-d**, 1.0 eq.) was added, and the mixture was stirred for 3 h at rt, followed by 3 h at 90 °C. After cooling to rt, the mixture was diluted with water and extracted with EtOAc (3x 10 mL). The combined organic layers were dried over Na_2SO_4, filtered, and concentrated *in vacuo*. The crude product was purified via reverse phase aFCC (water/ACN 8:2 to ACN 100%).

GP B - Benzimidazole Formation and Saponification: The respective amide derivative (**26a-e**; 1.0 eq.) was dissolved in 1 N HCl in 1,4-dioxane (0.05 M). The solution was heated to 80 °C under reflux overnight. 2 N NaOH(aq) (10 mL) was added, and the reaction mixture was heated to 80 °C under reflux for 2 h. After cooling to rt, 2 N HCl(aq) (20 mL) was added to precipitate the product. The pure product (**2**, **5**, **6**, **8**, **9**) was obtained by filtration and washing with ice-cold water (3x 5 mL) and EtOAc (3x 5 mL).

GP C - Buchwald-Hartwig Reaction: Methyl 4-aminobenzoate (**35**, 1.0 eq.), the respective sulfonamide (**34a-b**, 1.0 eq.) and cesium carbonate (4.0 eq.) were placed in a hot air-dried Schlenk flask, which was alternately flooded with Ar and evacuated three times after the addition. Degassed Toluene (0.05 M) was then added followed by tris(dibenzylideneacetone) dipalladium(0) (0.08 eq.) and 2-dicyclohexylphosphino-2,4,6-triisopropylbiphenyl (0.12 eq.) under Ar counter flow and stirring. The reaction mixture was stirred at 110 °C for 21 h. After cooling to rt, the mixture was filtered over a pad of silica and washed with MeOH. The solvents were removed under

reduced pressure, and the crude product was purified by aFCC (cyclohexane/EtOAc 98:2 to 85/15) and reverse phase aFCC (water/ACN 95:5 to 30:70).

GP D - Nucleophilic Aromatic Substitution with Phenols: The respective phenol derivative (**12a-b/e-g**, 1.1 eq.) was dissolved in dry DMF (0.1-0.25 M) and cooled to 0 °C. NaH (60% in mineral oil, 1.1 eq.) was added and the solution was allowed to warm to rt. After stirring at rt for 15 min, **11** or **23** (1.0 eq.) was added and the reaction mixture was heated to 80-90 °C under reflux for 3 h to overnight. After cooling to rt, water (10 mL) and EtOAc (50 mL) were added. The organic phase was washed with water (2x 30 mL) and brine (1x 30 mL), dried over $MgSO_4$, filtered, and the solvent was removed *in vacuo*. The crude product was purified *via* aFCC (cyclohexane) and reverse phase aFCC (water/ACN 7:3 to ACN 100%).

GP E - Aromatic Nitro Reduction: The respective nitro derivative (**13a,d**, 1.0 eq.) was dissolved in a mixture of MeOH/water (9:1, 0.2 M). Iron powder (10 eq.) and conc. HCl (10 % V/V) were added, and the mixture was stirred under argon atmosphere at 90 °C for 2 h. After cooling to rt, the mixture was quenched with sat. aq. $NaHCO_3$ solution and filtered over Celite. The filtrate was extracted with EtOAc (3x 10 mL), the combined organic layers were dried over Na_2SO_4, filtered, and concentrated *in vacuo*. The crude product was purified via aFCC (cyclohexane/EtOAc 9:1 to cyclohexane/EtOAc 6:4).

GP F - Nitro Reduction and Amide Coupling: The respective nitro derivative (**24a-e**, 1.0 eq.), iron powder (10 eq.) and NH_4Cl (1.0 eq.) were suspended in a mixture of EtOH/water (2:1, 0.1 M). The reaction mixture was heated to 80 °C under reflux overnight. After cooling to rt, the mixture was filtered through a plaque of Celite, which was washed using EtOAc (3x 30 mL). The filtrate was concentrated *in vacuo* and the residue (**25a-e**) was used for the following amide coupling reaction without further purification. The crude aniline derivative (**25a-e**, 1.0 eq.) and **19** (1.1 eq.) were dissolved in dry ACN (0.1 M). NMI (3.5 eq.) and TCFH (1.1 eq.) were added at 0 °C. The reaction mixture was stirred at 0 °C for 1 h and at rt overnight. The solvent was removed *in vacuo*, the residue was dissolved in EtOAc and washed with water (2x 30 mL) and brine (1x 30 mL). The organic layer was dried over $MgSO_4$, filtered, and concentrated *in vacuo*. The crude product was purified via aFCC (cyclohexane 100% to cyclohexane/EtOAc 1:1) and reverse phase aFCC (water/ACN 19:1 to ACN 100%).

GP G - Tertiary sulfonamide synthesis: NaH (60% in mineral oil, 1.2 eq.) was added to a solution of the respective sulfonamide (**32a-b**, 1.0 eq.) in dry DMF (0.2-0.3 M). The mixture was stirred at rt for 30 min before 4-bromobenzylbromide (**33**, 1.2 eq.) was added. The mixture was stirred at 80 °C until TLC indicated full conversion. After cooling to rt, the reaction was quenched with water, and the mixture was extracted with EtOAc (3x 10 mL). The combined organic layers were dried over Na_2SO_4, filtered, and concentrated *in vacuo*. The crude product was purified via aFCC (cyclohexane/EtOAc 98:2 to 85:15).

Synthesis and analytical characterization of ***A**, **C**, **1-3**, **5-10**, and **27-29***

3-((6-(3-Fluoro-4-methylphenoxy)-1-methyl-1*H*-1,3-benzodiazol-2-yl)methoxy)benzoic acid (A). The ester **20** (70.0 mg, 116 µmol, 1.0 eq.) was dissolved in 1,4-dioxane (1 mL, ~0.1 M), 2 M NaOH (0.5 mL) was added, and the mixture was stirred at 80 °C for 2 h. After cooling to rt, the mixture was acidified by the addition of 2 M HCl (1 mL) and the precipitated solid was collected by filtration. The pure product was obtained after washing with 2 M HCl (3x 1 mL) and water (2x 1 mL). **A** was obtained as off-white solid (69.0 mg, 100%). R_f = (cyclohexane/EtOAc 6:4) = 0.30. ^{1}H-NMR (400 MHz, DMSO-d_6): δ = 7.84 (d, *J* = 8.9 Hz, 1H), 7.75–7.69 (m, 1H), 7.69–7.62 (m, 2H), 7.52 (t, *J* = 7.8 Hz, 1H), 7.50–7.43 (m, 1H), 7.36–7.27 (m, 1H), 7.24 (dd, *J* = 8.9, 2.3 Hz, 1H), 6.89 (dd, *J* = 11.0, 2.5 Hz, 1H), 6.83 – 6.76 (m, 1H), 5.73 (s, 2H), 3.96 (s, 3H), 2.22 (d, *J* = 1.9 Hz, 3H) ppm. ^{13}C-NMR (101 MHz, DMSO-d_6): δ = 167.4, 161.4 (d, *J* = 244.3 Hz), 157.7, 156.8 (d, *J* = 10.8 Hz), 154.3, 150.4, 135.1, 132.9, 132.8 (d, *J* = 6.7 Hz), 130.5, 123.5, 120.2, 119.7, 119.5, 118.2, 117.7 115.9, 114.4 (d, *J* = 3.3 Hz), 106.1 (d, *J* = 25.5 Hz), 103.2, 61.9, 31.7, 14.0 ppm. HRMS (ESI+): *m/z* calculated 407.1407 for

$C_{23}H_{20}FN_2O_4$, found 407.1399 ([M+H]$^+$). qHNMR (400 MHz, DMSO-*d*$_6$, maleic acid as reference): purity = 97.5%.

4-((1-Methanesulfonylpiperidin-4-yl)amino)-*N*-(2-methylpropyl)-*N*-((2-(trifluoro methyl)phenyl)methyl)benzene-1-sulfonamide (C). In a Schlenk flask that was evacuated and backfilled with Argon (3x cycles), 4-iodo-*N*-(2-methylpropyl)-*N*-((2-(trifluoromethyl)phenyl)methyl)benzene-1-sulfonamide (**39b**, 548 mg, 1.10 mmol, 1.0 eq.), 4-amino-1-(methylsulfonyl)piperidine (263 mg, 1.43 mmol, 1.3 eq.), NaO*t*Bu (212 mg, 3.20 mmol, 2.9 eq.) and XPhos Pd G3 (93.3 mg, 110 µmol, 0.1 eq.) were dissolved in 1,4 dioxane (15.0 mL) and then stirred at 80 °C for 3 h. After cooling to rt, the mixture was diluted with water (25 mL) and extracted with EtOAc (3x 20 mL). The combined organic layers were dried over Na_2SO_4, filtered, and concentrated *in vacuo*. The crude product was purified via reverse phase aFCC (water/ACN 8:2 to ACN 100%). **C** was obtained as off-white solid (109 mg, 18%). R_f = (EtOAc) = 0.80. ^{1}H-NMR (400 MHz, $CDCl_3$): δ = 7.87 (d, *J* = 7.9 Hz, 1H), 7.60–7.46 (m, 4H), 7.29 (t, *J* = 7.6 Hz, 1H), 6.59–6.51 (m, 2H), 4.36 (s, 2H), 4.07 (d, *J* = 7.8 Hz, 1H), 3.79–3.69 (m, 2H), 3.49–3.37 (m, 1H), 2.91–2.80 (m, 4H), 2.76 (s, 3H), 2.16–2.11 (m, 1H), 1.62–1.55 (m, 1H), 1.55–1.52 (m, 1H), 1.48–1.33 (m, 1H), 0.68 (d, *J* = 6.6 Hz, 6H) ppm. ^{13}C-NMR (101 MHz, $CDCl_3$): δ = 150.0, 136.7, 132.0, 130.0, 129.5, 127.2, 126.9, 126.5 (q, *J* = 270 Hz), 126.1 (q, *J* = 31 Hz) 125.5 (q, *J* = 6.0 Hz), 112.2, 58.5, 50.0 (q, *J* = 3.3 Hz), 49.1, 44.8, 35.2, 31.7, 27.0, 20.1 ppm. HRMS (ESI+): *m/z* calculated 548.1865 for $C_{24}H_{33}F_3N_3O_4S_2$, found 548.1862 ([M+H]$^+$). qHNMR (400 MHz, DMSO-*d*$_6$, ethyl 4-(dimethylamino)benzoate as reference): purity = 97.5%.

5-((4-((1-Methyl-6-(4-methylphenoxy)-1*H*-1,3-benzodiazol-2-yl)methoxy)phenyl)methyl)-1,3-thiazolidine-2,4-dione (1). Synthesis was performed according to General Procedure A using **14a** (50.0 mg, 0.219 mmol, 1.0 eq.) and 2-(4-((2,4-dioxothiazolidin-5-yl)methyl)phenoxy)acetic acid (**17**, 61.6 mg, 0.219 mmol, 1.0 eq.). **1** was obtained as off-white solid (20.0 mg, 19%). R_f = (cyclohexane/EtOAc 6:4) = 0.19. ^{1}H-NMR (400 MHz, acetone-*d*$_6$): δ = 7.64 (dd, *J* = 8.7, 0.6 Hz, 1H), 7.32–7.22 (m, 2H), 7.22–7.06 (m, 5H), 7.01–6.85 (m, 3H), 5.41 (s, 2H), 4.79 (dd, *J* = 9.2, 4.2 Hz, 1H), 3.88 (s, 3H), 3.44 (dd, *J* = 14.2, 4.2 Hz, 1H), 3.15 (dd, *J* = 14.2, 9.3 Hz, 1H), 2.34–2.28 (m, 3H) ppm. ^{13}C-NMR (101 MHz, acetone-*d*$_6$): δ = 175.6, 171.4, 158.5, 157.1, 154.7, 151.0, 139.7, 138.0, 133.0, 131.4, 131.1, 130.6, 121.4, 118.9, 115.8, 115.5, 101.2, 63.9, 54.1, 37.9, 30.7, 20.6 ppm. HRMS (ESI+): *m/z* calculated 474.14877 for $C_{26}H_{24}N_3O_4S$, found 474.1476 ([M+H]$^+$). qHNMR (400 MHz, DMSO-*d*$_6$, ethyl 4-(dimethylamino)benzoate as reference): purity = 100%.

3-((4-Isopropoxy-1-methyl-6-(*p*-tolyloxy)-1*H*-benzo[*d*]imidazol-2-yl)methoxy)benzoic acid (2). **2** was synthesized according to General Procedure B using amide **26a** (33.5 mg, 0.0700 mmol, 1.0 eq.), 2 N HCl in 1,4-dioxane (4.0 mL), 2 N NaOH(aq) (10 mL) and 2 N HCl(aq) (20 mL). **2** (15.0 mg, 48%) was obtained as a colorless solid. R_f (cyclohexane/EtOAc 6:4 + 1% AcOH) = 0.35. ^{1}H-NMR (500 MHz, acetone-*d*$_6$) δ = 7.81 (dd, *J* = 2.7, 1.5 Hz, 1H), 7.75–7.67 (m, 2H), 7.48 (t, *J* = 8.0 Hz, 1H), 7.25–7.19 (m, 2H), 7.01–6.95 (m, 2H), 6.93 (d, *J* = 1.9 Hz, 1H), 6.80 (d, *J* = 1.9 Hz, 1H), 6.07 (s, 2H), 4.94 (hept, *J* = 6.0 Hz, 1H), 4.12 (s, 3H), 2.33 (s, 3H), 1.40 (d, *J* = 6.0 Hz, 6H) ppm. ^{13}C-NMR (126 MHz, acetone-*d*$_6$) δ = 167.0, 159.4, 158.4, 158.3, 155.6, 148.7, 148.3, 136.5, 134.3, 133.2, 131.3, 130.9, 124.3, 120.4, 119.8, 117.3, 103.6, 93.7, 73.4, 60.2, 32.3, 22.1, 20.7 ppm. HRMS (ESI+): *m/z* calculated 485.1473 for $C_{26}H_{26}O_5N_2K$, found 485.1470 ([M+K]$^+$). qHNMR (400 MHz, DMSO-*d*$_6$, ethyl 4-(dimethylamino)benzoate as reference): purity = 95.1%.

5-((4-((1-Methyl-6-(3-(trifluoromethyl)phenoxy)-1*H*-1,3-benzodiazol-2-yl)methoxy)phenyl)methyl)-1,3-thiazolidine-2,4-dione (3). Synthesis was performed according to General Procedure A using **14b** (140 mg, 496 µmol, 1.0 eq.) and 2-(4-((2,4-dioxothiazolidin-5-yl)methyl)phenoxy)acetic acid (**17**, 140 mg, 496 µmol, 1.0 eq.). **3** was obtained as off-white solid (5.00 mg, 2.0%). R_f = (cyclohexane/EtOAc 6:4) = 0.60. ^{1}H-NMR (400 MHz, DMSO-*d*$_6$): δ = 12.04 (s, 1H), 7.72 (d, *J* = 8.6 Hz, 1H), 7.63 – 7.55 (m, 1H), 7.49–7.41 (m, 2H), 7.32–7.17 (m, 4H), 7.12–7.05 (m, 2H), 7.05–6.95 (m, 1H), 5.39 (s, 2H), 4.87 (dd, *J* = 9.2, 4.3 Hz, 1H), 3.83 (s, 3H), 3.07 (dd, *J* = 14.2, 9.2 Hz, 1H), 2.68 (dd, *J* = 14.2, 9.3 Hz, 1H) ppm. ^{13}C-NMR (126 MHz, DMSO-*d*$_6$): δ = 176.5, 172.3, 159.4, 157.4, 151.4, 151.1, 139.5, 137.2, 131.8, 131.2, 130.9, 130.1, 129.1–122.8 (m), 121.5, 121.1, 119.7-

119.3 (m), 115.6, 115.3, 114.2–113.7 (m), 102.9, 63.0, 53.5, 36.8, 30.7 ppm. HRMS (ESI+): *m/z* calculated 528.1205 for $C_{26}H_{21}F_3N_3O_4S$, found 528.1190 ($[M+H]^+$). qHNMR (400 MHz, DMSO-*d6*, ethyl 4-(dimethylamino)benzoate as reference): purity = 100%.

3-((6-(3,5-Dimethylphenoxy)-4-isopropoxy-1-methyl-1*H*-benzo[*d*]imidazol-2-yl)methoxy)benzoic acid (5). **5** was synthesized according to General Procedure B using amide **26b** (325 mg, 0.660 mmol, 1.0 eq.), 1 N HCl in 1,4-dioxane (8 mL), 2 N NaOH(aq) (6.0 mL) and 2 N HCl(aq) (10 mL). **5** (235 mg, 77%) was obtained as a colorless solid. R_f (cyclohexane/EtOAc 6:4 + 1% AcOH) = 0.36. ^{1}H-NMR (500 MHz, DMSO-*d6*) δ = 7.67 (dd, *J* = 2.7, 1.5 Hz, 1H), 7.62 (dt, *J* = 7.5, 1.2 Hz, 1H), 7.49 (t, *J* = 7.9 Hz, 1H), 7.40 (ddd, *J* = 8.2, 2.7, 1.1 Hz, 1H), 6.98 (d, *J* = 2.0 Hz, 1H), 6.77 (s, 1H), 6.73 (s, 1H), 6.64 (d, *J* = 1.5 Hz, 2H), 5.58 (s, 2H), 4.99 (hept, *J* = 6.0 Hz, 1H), 3.87 (s, 3H), 2.24 (s, 6H), 1.33 (d, *J* = 6.0 Hz, 6H) ppm. ^{13}C-NMR (101 MHz, DMSO-*d6*) δ = 166.9, 159.0, 157.5, 153.1, 149.2, 148.4, 148.3, 136.4, 132.4, 131.5, 130.0, 122.8, 121.3, 119.8, 118.7, 116.2, 115.3, 115.1, 110.3, 102.0, 95.3, 71.3, 61.3, 31.1, 21.7 ppm. HRMS (ESI+): *m/z* calculated 483.1890 for $C_{27}H_{28}O_5N_2Na$, found 483.1888 ($[M+Na]^+$). qHNMR (400 MHz, DMSO-*d6*, ethyl 4-(dimethylamino)benzoate as reference): purity = 96.2%.

3-((4-Isopropoxy-1-methyl-6-(3-(trifluoromethoxy)phenoxy)-1*H*-benzo[*d*]imidazol-2-yl)methoxy)benzoic acid (6). **6** was synthesized according to General Procedure B using amide **26c** (170 mg, 0.310 mmol, 1.0 eq.), 1 N HCl in 1,4-dioxane (9.0 mL), 2 N NaOH(aq) (10 mL) and 2 N HCl(aq) (20 mL). **6** (140 mg, 88%) was obtained as a colorless solid. R_f (cyclohexane/EtOAc 6:4 + 1% AcOH) = 0.36. ^{1}H-NMR (400 MHz, DMSO-*d6*) δ = 7.68 (dd, *J* = 2.7, 1.4 Hz, 1H), 7.62 (dt, *J* = 7.6, 1.2 Hz, 1H), 7.50 (t, *J* = 8.2 Hz, 1H), 7.49 (t, *J* = 8.1 Hz, 1H), 7.41 (ddd, *J* = 8.3, 2.7, 1.1 Hz, 1H), 7.16 (d, *J* = 1.9 Hz, 1H), 7.12 (ddq, *J* = 8.3, 2.1, 1.1 Hz, 1H), 7.03 (ddd, *J* = 8.3, 2.4, 0.9 Hz, 1H), 7.00 (td, *J* = 2.2, 1.0 Hz, 1H), 6.81 (d, *J* = 1.9 Hz, 1H), 5.58 (s, 2H), 5.00 (hept, *J* = 6.0 Hz, 1H), 3.89 (s, 3H), 1.33 (d, *J* = 6.0 Hz, 6H) ppm. ^{13}C-NMR (101 MHz, DMSO-*d6*) δ = 166.9, 159.0, 157.5, 153.1, 149.2, 148.4, 148.3, 136.4, 132.4, 131.5, 130.0, 122.8, 120.0 (q, *J* = 257.0 Hz), 119.8, 116.2, 115.3, 115.1, 111.9, 110.3, 102.0, 95.3, 71.3, 61.3, 31.1, 21.7ppm. HRMS (ESI+): *m/z* calculated 539.1400 for $C_{26}H_{23}O_6N_2F_3Na$, found 539.1392 ($[M+Na]^+$). qHNMR (400 MHz, Acetone-*d6*, ethyl 4-(dimethylamino)benzoate as reference): purity = 95.3%.

5-(4-((6-((2,3-Dihydrobenzofuran-6-yl)oxy)-1-methyl-1*H*-benzo[*d*]imidazol-2-yl)methoxy)benzyl)thiazolidine-2,4-dione (7). Synthesis was performed according to General Procedure A using **14c** (140 mg, 0.546 mmol, 1.0 eq.) and 2-(4-((2,4-dioxothiazolidin-5-yl)methyl)phenoxy)acetic acid (**17**, 154 mg, 0.546 mmol, 1.0 eq.). **7** was obtained as an off-white solid (60.0 mg, 22%). R_f = (cyclohexane/EtOAc 6:4) = 0.25. ^{1}H-NMR (400 MHz, DMSO-*d6*): δ = 12.02 (s, 1H), 7.64 (d, *J* = 8.7 Hz, 1H), 7.27 (d, *J* = 2.3 Hz, 1H), 7.23–7.13 (m, 3H), 7.11–7.04 (m, 2H), 6.91 (dd, *J* = 8.7, 2.3 Hz, 1H), 6.46–6.38 (m, 2H), 5.37 (s, 2H), 4.87 (dd, *J* = 9.2, 4.3 Hz, 1H), 4.56 (t, *J* = 8.7 Hz, 2H), 3.80 (s, 3H), 3.18-2.99 (m, 4H) ppm. ^{13}C-NMR (126 MHz, DMSO-*d6*): δ = 176.5, 172.4, 161.4, 158.6, 157.4, 153.1, 150.6, 138.7, 137.1, 130.9, 130.1, 125.8, 122.1, 120.7, 115.3, 115.1, 110.0, 101.5, 100.1, 72.3, 63.0, 53.6, 36.8, 30.6, 28.9 ppm. HRMS (ESI+): *m/z* calculated 502.1437 for $C_{27}H_{24}N_3O_5S$, found 502.1422 ($[M+H]^+$). qHNMR (400 MHz, DMSO-*d6*, ethyl 4-(dimethylamino)benzoate as reference): purity = 96.4%.

3-((4-Isopropoxy-1-methyl-6-(3-(trifluoromethyl)phenoxy)-1*H*-benzo[*d*]imidazol-2-yl)methoxy)benzoic acid (8). **8** was synthesized according to General Procedure B using amide **26d** (150 mg, 0.282 mmol, 1.0 eq.), 1 N HCl in 1,4-dioxane (8.0 mL), 2 N NaOH(aq) (6.0 mL) and 2 N HCl(aq) (10 mL). **8** (73.2 mg, 52%) was obtained as a colorless solid. R_f (cyclohexane/EtOAc 6:4 + 1% AcOH) = 0.23. ^{1}H-NMR (500 MHz, DMSO-*d6*) δ = 7.68 (dd, *J* = 2.7, 1.4 Hz, 1H), 7.65–7.59 (m, 2H), 7.49 (t, *J* = 7.9 Hz, 2H), 7.41 (ddd, *J* = 8.3, 2.7, 1.1 Hz, 1H), 7.34–7.28 (m, 2H), 7.16 (d, *J* = 2.0 Hz, 1H), 6.82 (s, 1H), 5.58 (s, 2H), 5.01 (hept, *J* = 6.0 Hz, 1H), 3.89 (s, 3H), 1.33 (d, *J* = 6.0 Hz, 6H) ppm. ^{13}C-NMR (126 MHz, DMSO-*d6*) δ = 166.9, 158.3, 157.5, 153.2, 148.4, 148.3, 136.4, 132.4, 131.4, 130.6 (q, *J* = 31.8 Hz), 130.0, 123.8 (q, *J* = 272.3 Hz), 122.8, 121.4, 119.8, 119.5 (q, *J* = 4.1 Hz), 115.3, 113.8 (q, *J* = 3.8 Hz), 109.6 (q, *J* = 33.1 Hz), 102.0, 95.3, 71.3, 61.3, 31.1, 21.7 ppm. HRMS (ESI+):

m/z calculated 523.1451 for $C_{26}H_{23}F_3O_5N_2Na$, found 523.1446 ($[M+Na]^+$). qHNMR (400 MHz, DMSO-*d6*, ethyl 4-(dimethylamino)benzoate as reference): purity = 97.7%.

3-((4-Isopropoxy-1-methyl-6-(*m*-tolyloxy)-1*H*-benzo[*d*]imidazol-2-yl)methoxy)benzoic acid (9). **9** was synthesized according to General Procedure B using amide **26e** (280 mg, 0.585 mmol, 1.0 eq.), 1 N HCl in 1,4-dioxane (7.5 mL), 2 N NaOH(aq) (10 mL) and 2 N HCl(aq) (20 mL). **9** (182 mg, 70%) was obtained as a colorless solid. R_f (cyclohexane/EtOAc 6:4 + 1% AcOH) = 0.35. ^{1}H-NMR (400 MHz, acetone-*d6*) δ = 7.80 (dd, *J* = 2.7, 1.4 Hz, 1H), 7.72–7.63 (m, 2H), 7.50–7.44 (m, 1H), 7.27 (t, *J* = 7.9 Hz, 1H), 6.98 (ddt, *J* = 7.5, 1.7, 0.9 Hz, 1H), 6.94 (d, *J* = 1.9 Hz, 1H), 6.90 (d, *J* = 2.3 Hz, 1H), 6.85 (dd, *J* = 8.2, 2.5 Hz, 1H), 6.75 (d, *J* = 1.9 Hz, 1H), 5.99 (s, 2H), 4.98 (hept, *J* = 6.0 Hz, 1H), 4.10 (s, 3H), 2.31 (s, 3H), 1.39 (d, *J* = 6.1 Hz, 6H) ppm. ^{13}C-NMR (126 MHz, acetone-*d6*) δ = 167.1, 158.4, 158.2, 157.5, 149.0, 148.4, 141.0, 136.9, 133.2, 130.8, 130.6, 127.2, 125.3, 124.1, 120.4, 120.1, 117.2, 116.5, 103.7, 94.3, 73.2, 60.7, 32.1, 22.2, 21.3 ppm. HRMS (ESI+): *m/z* calculated 469.1734 for $C_{26}H_{26}O_5N_2Na$, found 469.1729 ($[M+Na]^+$). qHNMR (400 MHz, DMSO-*d6*, ethyl 4-(dimethylamino)benzoate as reference): purity = 95.3%.

5-((4-((6-(3-Fluoro-4-methylphenoxy)-1-methyl-1*H*-1,3-benzodiazol-2-yl)methoxy)phenyl)methyl)-1,3-thiazolidine-2,4-dione (10). Synthesis was performed according to General Procedure A using **14d** (54.0 mg, 0.220 mmol, 1.0 eq.) and 2-(4-((2,4-dioxothiazolidin-5-yl)methyl)phenoxy)acetic acid (**17**, 62.0 mg, 0.220 mmol, 1.0 eq.). **10** was obtained as an off-white solid (10.0 mg, 10%). R_f = (cyclohexane/EtOAc 6:4) = 0.29. ^{1}H-NMR (500 MHz, acetone-*d6*) δ = 8.66 (s, 1H), 7.33–7.28 (m, 2H), 7.25–7.19 (m, 1H), 7.15 (dd, *J* = 8.4, 3.8 Hz, 1H), 7.08–7.02 (m, 2H), 6.77–6.69 (m, 2H), 6.34 (d, *J* = 2.5 Hz, 1H), 6.25 (dd, *J* = 8.4, 2.6 Hz, 1H), 4.82 (dd, *J* = 9.0, 4.2 Hz, 1H), 4.65 (s, 2H), 3.46 (dd, *J* = 14.1, 4.3 Hz, 1H), 3.20 (dd, *J* = 14.2, 9.0 Hz, 1H), 2.73 (d, *J* = 4.5 Hz, 3H), 2.21 (d, *J* = 2.0 Hz, 3H) ppm. ^{13}C-NMR (126 MHz, acetone-*d6*) δ = 175.6, 171.4, 167.9, 162.3 (d, *J* = 243.9 Hz), 158.0, 157.9, 157.1, 147.4, 132.8 (d, *J* = 6.6 Hz), 131.6, 130.7, 128.7, 119.6, 119.5 (d, *J* = 17.3 Hz), 115.7, 114.7 (d, *J* = 3.3 Hz), 106.7, 106.2 (d, *J* = 25.5 Hz), 102.5, 68.3, 54.1, 37.8, 30.4, 13.8 (d, *J* = 3.3 Hz) ppm. HRMS (ESI+): *m/z* calculated 492.1394 for $C_{26}H_{23}FN_3O_4S$, found 492.1381 ($[M+H]^+$). qHNMR (400 MHz, DMSO-*d6*, ethyl 4-(dimethylamino)benzoate as reference): purity = 98.4%.

Methyl 4-((4-(((*N*-(*tert*-butyl)-1-phenylmethyl)sulfonamido)methyl)phenyl)amino)benzoate (27). Synthesis of **27** was performed according to General Procedure C using methyl 4-aminobenzoate (**35**, 36.7 mg, 0.238 mmol, 1.1 eq.), **34a** (90.0 mg, 0.227 mmol, 1.0 eq.), cesium carbonate (296 mg, 0.908 mmol, 4.0 eq.), tris(dibenzylidene-acetone)dipalladium(0) (16.5 mg, 18.0 µmol, 0.08 eq.) and 2-dicyclohexyl-phosphino-2',4',6'-triisopropylbiphenyl (13.1 mg, 27.0 *µ*mol, 0.12 eq.) in degassed toluene (4.0 mL). During filtration, the crude product was washed with MeOH. **27** (41.0 mg, 39%) was obtained as a pale yellow solid. R_f = (cyclohexane/EtOAc; 8:2) = 0.29. ^{1}H-NMR (400 MHz, $CDCl_3$): δ = 7.92–7.87 (m, 2H), 7.45–7.35 (m, 5H), 7.32–7.28 (m, 2H), 7.09–7.05 (m, 2H), 6.96–6.92 (m, 2H), 4.31 (s, 2H), 4.04 (s, 2H), 3.87 (s, 3H), 1.45 (s, 9H) ppm. ^{13}C-NMR (101 MHz, $CDCl_3$): δ = 167.1, 148.1, 139.9, 134.2, 131.6, 131.0, 129.8, 129.1, 128.83, 128.75, 121.2, 120.3, 114.7, 62.0, 60.1, 51.9, 50.6, 30.9 ppm. HRMS (ESI+) m/z calculated 489.1819 for $C_{26}H_{30}O_4N_2NaS$, found 489.1815 ($[M+Na]^+$). qHNMR (101 MHz, $CDCl_3$, ethyl 4-(dimethylamino)benzoate as reference): purity = 99.8%

Methyl 4-((4-(((*N*-isobutyl-1-phenylmethyl)sulfonamido)methyl)phenyl)amino)benzoate (28). Synthesis of **28** was performed according to General Procedure C using methyl 4-aminobenzoate (**35**, 29.5 mg, 0.191 mmol, 1.1 eq.), **34b** (72.0 mg, 0.182 mmol, 1.0 eq.), cesium carbonate (237 mg, 0.728 mmol, 4.0 eq.), tris(dibenzylidene-acetone)dipalladium(0) (13.3 mg, 14.6 µmol, 0.08 eq.) and 2-dicyclohexyl-phosphino-2',4',6'-triisopropylbiphenyl (10.6 mg, 21.2 µmol, 0.12 eq.) in degassed toluene (4.0 mL). During filtration, the crude product was washed with MeOH. **28** (55.2 mg, 65%) was obtained as a pale yellow solid. R_f = (cyclohexane/EtoAC 8:2) = 0.30. ^{1}H-NMR (400 MHz, $CDCl_3$): δ = 7.94–7.88 (m, 2H), 7.40–7.32 (m, 5H), 7.32–7.27 (m, 2H), 7.15–7.09 (m, 2H), 7.01–6.95 (m, 2H), 4.20 (s, 2H), 4.15 (s, 2H), 3.87 (s, 3H), 2.84 (d, *J* = 7.5 Hz, 2H), 1.73–1.61 (hept, *J* = 6.9 Hz, 1H), 0.79 (d, *J* = 6.7 Hz, 6H) ppm. ^{13}C-NMR (101 MHz, $CDCl_3$): δ = 167.0, 147.8, 140.8, 131.6, 131.4, 130.9, 130.1, 129.2, 128.9, 128.8, 121.6, 120.1, 115.0, 58.5, 56.7, 52.6, 51.9,

27.1, 20.1 ppm. HRMS (ESI+): *m/z* calculated 489.1819 for $C_{26}H_{30}O_4N_2NaS$, found 489.1814 ($[M+Na]^+$). qHNMR (400 MHz, DMSO-d_6, ethyl 4-(dimethylamino)benzoate as reference): purity = 98.7%.

***N-tert*-Butyl-4-[(1-methanesulfonylpiperidin-4-yl)amino]-*N*-{[2-(trifluoromethyl) phenyl]methyl}benzene-1-sulfonamide (29)**. In a Schlenk flask that was evacuated and backfilled with Argon (3x cycles), *N*-tert-butyl-4-iodo-*N*-((2-(trifluoromethyl)phenyl)methyl)benzene-1-sulfonamide (**40a**, 328 mg, 0.660 mmol, 1.0 eq.), 4-amino-1-(methylsulfonyl)piperidine (158 mg, 0.857 mmol, 1.3 eq.), NaO*t*Bu (127 mg, 1.32 mmol, 2.9 eq.) and XPhos Pd G3 (55.8 mg, 66.0 µmol, 0.1 eq.) were dissolved in 1,4 dioxane (10.0 mL), and the mixture was stirred at 80 °C for 3 h. After cooling to rt, the mixture was diluted with water (25 mL) and extracted with EtOAc (3x 20 mL). The combined organic layers were dried over Na_2SO_4, filtered, and concentrated *in vacuo.* The crude product was purified via reverse phase aFCC (water/ACN 8:2 to ACN 100%). **29** was obtained as a transparent solid (88.4 mg, 25%). R_f = (EtOAc) = 0.80. ^{1}H-NMR (400 MHz, $CDCl_3$): δ = 7.97 (d, *J* = 7.9 Hz, 1H), 7.68–7.60 (m, 2H), 7.63–7.53 (m, 2H), 7.33 (t, *J* = 7.6 Hz, 1H), 6.63–6.54 (m, 2H), 4.80 (s, 2H), 4.09 (d, *J* = 7.8 Hz, 1H), 3.85–3.75 (m, 2H), 2.96–2.85 (m, 2H), 2.82 (s, 3H), 2.21–2.12 (m, 2H), 1.67–1.60 (m, 1H), 1.60–1.55 (m, 1H), 1.26 (s, 9H) ppm. ^{13}C-NMR (101 MHz, $CDCl_3$): δ = 149.8, 139.5, 132.2, 130.3, 129.7, 129.1, 126.8, 126.5 (q, *J* = 270 Hz), 126.1 (q, *J* = 31 Hz), 125.6 (q, *J* = 5.8 Hz), 112.2, 59.9, 49.2, 47.3 (q, *J* = 3.3 Hz), 45.0, 35.3, 31.8, 30.0 ppm. HRMS (ESI+): *m/z* calculated 548.1865 for $C_{24}H_{33}F_3N_3O_4S_2$, found 548.1858 ($[M+H]^+$). qHNMR (400 MHz, DMSO-d_6, ethyl 4-(dimethylamino)benzoate as reference): purity = 96.2%.

*Synthesis and analytical characterization of intermediates **13a-d, 14a-d, 15-17, 19, 22, 23, 24a-e, 26a-e, 32a-b, 34a-b, 37a-b, 39a-b***

***N*-Methyl-2-nitro-5-(*p*-tolyloxy)aniline (13a).** Synthesis was performed according to General Procedure D using **11** (540 mg, 2.89 mmol, 1.0 eq.) and *p*-cresol (**12a**, 344 mg, 3.18 mmol, 1.1 eq.). **13a** was obtained as a yellow solid (700 mg, 93%). R_f = (cyclohexane/EtOAc 6:4) = 0.19. ^{1}H-NMR (400 MHz, $CDCl_3$): δ = 8.19 (s, 1H), 8.14 (d, *J* = 9.4 Hz, 1H), 7.25–7.17 (m, 2H), 7.04–6.93 (m, 2H), 6.24 (d, *J* = 2.4 Hz, 1H), 6.20 (dd, *J* = 9.4, 2.5 Hz, 1H), 2.90 (d, *J* = 5.1 Hz, 3H), 2.38 (s, 3H) ppm. ^{13}C-NMR (101 MHz, $CDCl_3$): δ = 165.2, 152.3, 148.6, 135.1, 130.7, 129.5, 127.5, 120.7, 105.7, 99.1, 29.8, 21.0 ppm. (APCI+): *m/z* 258.8 ($[M+H]^+$).

5-(3-Fluoro-4-methylphenoxy)-*N*-methyl-2-nitroaniline (13d). Synthesis was performed according to General Procedure D using **11** (540 mg, 2.89 mmol, 1.0 eq.) and 3-fluoro-4-methylphenol **12d** (402 mg, 3.18 mmol, 1.1 eq.). **13d** was obtained as yellow solid (700 mg, 93%). R_f = (cyclohexane/EtOAc 6:4) = 0.20. ^{1}H-NMR (400 MHz, $CDCl_3$): δ = 8.22–8.11 (m, 1H), 7.25–7.15 (m, 1H), 6.83–6.75 (m, 2H), 6.28 (d, *J* = 2.5 Hz, 1H), 6.21 (dd, *J* = 9.4, 2.5 Hz, 1H), 2.92 (d, *J* = 5.1 Hz, 3H), 2.31–2.26 (m, 3H) ppm. ^{13}C-NMR (101 MHz, $CDCl_3$): δ = 164.0, 161.3 (d, *J* = 247.2 Hz), 153.2 (d, *J* = 10.3 Hz), 148.2, 131.9 (d, *J* = 6.6 Hz), 129.3, 127.6, 121.5 (d, *J* = 17.3 Hz), 115.7 (d, *J* = 3.5 Hz), 107.8 (d, *J* = 25.1 Hz), 105.4, 99.3, 29.5, 13.9 (d, *J* = 3.1 Hz). (APCI+): *m/z* 276.4 ($[M+H]^+$).

N^1-Methyl-5-(*p*-tolyloxy)benzene-1,2-diamine (14a). Synthesis was performed according to General Procedure E using **13a** (700 mg, 2.71 mmol, 1.0 eq.). **14a** was obtained as purple oil (100 mg, 16%). R_f = (cyclohexane/EtOAc 6:4) = 0.30. ^{1}H-NMR (400 MHz, $CDCl_3$): δ = 7.15–7.07 (m, 2H), 6.94–6.86 (m, 2H), 6.68 (d, *J* = 8.2 Hz, 1H), 6.41 (d, *J* = 2.6 Hz, 1H), 6.31 (dd, *J* = 8.2, 2.6 Hz, 1H), 2.83 (s, 3H), 2.33 (s, 3H) ppm. ^{13}C-NMR (101 MHz, $CDCl_3$): δ = 156.4, 151.8, 131.5, 130.2, 129.9, 129.1, 118.4, 117.6, 117.3, 115.2, 108.1, 103.0, 30.8, 20.7 ppm. (APCI+): *m/z* 229.2 ($[M+H]^+$).

N^1-Methyl-5-(3-(trifluoromethyl)phenoxy)benzene-1,2-diamine (14b). Synthesis was performed according to General Procedure D using **11** (540 mg, 2.89 mmol, 1.0 eq.) and *m*-trifluoromethyl phenol **12b** (387 µL, 3.18 mmol, 1.1 eq.). **13b** was obtained as yellow solid and used for the next step without further purification. The crude *N*-methyl-2-nitro-5-(3-(trifluoromethyl)phenoxy)aniline (**13b**, 460 mg, 1.47 mmol) was dissolved in EtOH (5.0 mL) followed by addition of iron (III) acetylacetonate (52.7 mg, 0.145 mmol, 0.06 eq.) and hydrazine hydrate (270 µL, 4.34 mmol, 1.8 eq.). The mixture was stirred at 80 °C overnight. After cooling to rt, the mixture was quenched with sat. aq. $NaHCO_3$ solution and filtered over Celite. The filtrate was extracted with EtOAc (3x 10 mL), the combined organic layers were dried over Na_2SO_4, filtered, and concentrated *in vacuo*. **14b** was obtained as purple oil (140 mg, 21%). R_f = (cyclohexane/EtOAc 6:4) = 0.20. ^{1}H-NMR (400 MHz, $CDCl_3$): δ = 7.30 (t, *J* = 8.0 Hz, 1H), 7.22–7.14 (m, 1H), 7.14-7.11 (m, 1H), 7.08–7.01 (m, 1H), 6.63 (d, *J* = 8.1 Hz, 1H), 6.30 (d, *J* = 2.6 Hz, 1H), 6.26 (dd, *J* = 8.1, 2.6 Hz, 1H), 3.57 (s, 1H), 3.15 (s, 2H), 2.75 (s, 3H) ppm. ^{13}C-NMR (126 MHz, $CDCl_3$): δ = 159.3, 150.1, 141.1, 131.9 (q, *J* = 32.4 Hz), 130.2, 130.0, 127.4–120.5 (m), 120.3, 118.4 (q, *J* = 3.8 Hz), 117.3, 113.9 (q, *J* = 3.9 Hz), 108.9, 103.3, 30.8 ppm. (APCI+): *m/z* 283.4 ($[M+H]^+$).

5-((2,3-Dihydrobenzofuran-6-yl)oxy)-N^1-methylbenzene-1,2-diamine (14c). Synthesis was performed according to General Procedure D_1 using **11** (311 mg, 1.67 mmol, 1.0 eq.) and 2,3-dihydro-1-benzofuran-6-ol (**12c**, 250 mg, 1.84 mmol, 1.1 eq.). **13c** was obtained as yellow oil and used for the next step without further purification. The crude 5-((2,3-dihydrobenzofuran-6-yl)oxy)-*N*-methyl-2-nitroaniline (**13c**, 480 mg, 1.68 mmol) was dissolved in EtOH (5.0 mL) followed by addition of iron (III) acetylacetonate (36.6 mg, 0.101 mmol, 0.06 eq.) and hydrazine hydrate (188 µL, 3.02 mmol, 1.8 eq.). The mixture was stirred at 80 °C overnight. After cooling to rt, the mixture was quenched with sat. aq. $NaHCO_3$ solution and filtered over Celite. The filtrate was extracted with EtOAc (3x 10 mL), the combined organic layers were dried over Na_2SO_4, filtered, and concentrated *in vacuo*. **14c** was obtained as purple oil (140 mg, 33%). R_f = (cyclohexane/EtOAc 8:2) = 0.20. ^{1}H-NMR (400 MHz, $CDCl_3$): δ = 7.04–6.95 (m, 1H), 6.58 (d, *J* = 8.2 Hz, 1H), 6.42–6.33 (m, 2H), 6.31 (d, *J* = 2.6 Hz, 1H), 6.24 (dd, *J* = 8.2, 2.6 Hz, 1H), 4.50 (t, *J* = 8.7 Hz, 2H), 3.12–3.03 (m, 2H), 2.74 (s, 3H) ppm. ^{13}C-NMR (126 MHz,

$CDCl_3$): δ = 161.2, 159.3, 151.4, 140.9, 129.4, 124.8, 120.4, 117.2, 109.4, 108.5, 103.2, 99.8, 72.1, 30.8, 29.1 ppm. (APCI+): *m/z* 257.2 ($[M+H]^+$).

5-(3-Fluoro-4-methylphenoxy)-*N*[1]-methylbenzene-1,2-diamine (14d). Synthesis was performed according to General Procedure E using **13d** (700 mg, 2.71 mmol, 1.0 eq.). **14d** was obtained as purple oil (181 mg, 13%). R_f = (cyclohexane/EtOAc 6:4) = 0.20. ^{1}H-NMR (400 MHz, $CDCl_3$): δ = 7.08–7.02 (m, 1H), 6.69–6.63 (m, 3H), 6.37 (d, *J* = 2.6 Hz, 1H), 6.32 (dd, *J* = 8.2, 2.6 Hz, 1H), 2.81 (s, 3H), 2.21 (d, *J* = 2.0 Hz, 3H) ppm. ^{13}C-NMR (101 MHz, $CDCl_3$): δ = 141.0, 140.3, 132.2, 131.4 (d, *J* = 6.8 Hz), 129.7, 126.0, 118.0 (d, *J* = 17.6 Hz), 117.2, 112.7 (d, *J* = 3.4 Hz), 108.6, 104.7 (d, *J* = 25.7 Hz), 103.2, 30.8, 13.9 ppm. (APCI+): *m/z* 246.7 ($[M+H]^+$).

(*E*)-2-(4-((2,4-Dioxothiazolidin-5-ylidene)methyl)phenoxy)acetic acid (16). To a stirred solution of 2-(4-formylphenoxy)acetic acid (**15**, 2.20 g, 12.2 mmol, 1.0 eq.) and thiazolidine-2,4-dione (1.43 g, 12.2 mmol, 1.0 eq.) in EtOH (36 mL), aq. KOH solution (40%, 18 mL) was added in portions at rt over 10 min. The mixture was stirred at rt overnight. After completion of the reaction according to TLC, the reaction mixture was poured into crushed ice and acidified with conc. HCl (pH 3). The resulting precipitate was filtered off and washed with 2 M HCl (3x 5 mL) and water (3 x 5 mL). For further purification of the crude product was performed by reverse phase aFCC (water/ACN 8:2 to ACN 100%). **16** was obtained as a transparent solid (3.40 g, 99%). R_f = (EtOAc + Acetic acid 2%) = 0.30. ^{1}H-NMR (400 MHz, DMSO-*d*$_6$): δ = 12.56 (s, 1H), 7.76 (s, 1H), 7.60–7.52 (m, 2H), 7.13–7.04 (m, 2H), 4.78 (s, 2H) ppm. ^{13}C-NMR (101 MHz, DMSO-*d*$_6$): δ = 170.2, 168.5, 167.9, 159.9, 132.5, 132.2, 126.4, 121.1, 115.9, 65.0 ppm. (APCI+): *m/z* 280.1 ($[M+H]^+$).

2-(4-((2,4-Dioxothiazolidin-5-yl)methyl)phenoxy)acetic acid (17). **16** (3.40 g, 12.2 mmol, 1.0 eq.) was added to a mixture of water (13.5 mL), methanol (9.0 mL), and NaOH (1 M solution, 9 mL), and the resultant mixture was stirred for 15 min at rt before 0.3 mL of a solution of $CoCl_2$−DMG complex (42 mg of $CoCl_2 \cdot 6H_2O$ and 250 mg of dimethylglyoxime in 5 mL of DMF) were added. After stirring for another 15 min, $NaBH_4$ (0.58 g, 15.2 mmol, 1.25 eq.) in water (13.5 mL) was added in a single portion. The resulting yellow mixture was warmed to 35 °C and stirred for 3 h, resulting in a clear solution. After cooling to rt, the mixture was brought to pH 2 by the addition of aq. HCl (2 M, 20 mL) and extracted with Et_2O (3x 10 mL). The combined organic layers were dried over Na_2SO_4, filtered, and concentrated *in vacuo*. For further purification the crude product was performed via reverse phase aFCC (water/ACN 8:2 to ACN 100%). **16** was obtained as a transparent solid (1.37 g, 40%). R_f = (EtOAc + acetic acid 2%) = 0.30. ^{1}H-NMR (400 MHz, DMSO-*d*$_6$): δ = 7.19–7.11 (m, 2H), 6.88–6.78 (m, 2H), 4.84 (dd, *J* = 9.2, 4.3 Hz, 1H), 4.57 (s, 2H), 3.31 (dd, *J* = 14.2, 4.3 Hz, 1H), 3.03 (dd, *J* = 14.2, 9.3 Hz, 1H) ppm. ^{13}C-NMR (101 MHz, DMSO-*d*$_6$): δ = 176.9, 172.6, 171.0, 157.4, 130.7, 129.6, 114.8, 65.4, 53.8, 36.9 ppm. (APCI+): *m/z* 282.4 ($[M+H]^+$).

2-(3-(Methoxycarbonyl)phenoxy)acetic acid (19). Methyl 3-hydroxybenzoate (**18**, 5.00 g, 32.9 mmol, 1.0 eq.), K_2CO_3 (9.99 g, 72.3 mmol, 2.2 eq.) and *tert*-butyl bromoacetate (4.85 mL, 32.9 mmol, 1.0 eq.) were dissolved in DMF (25 mL) and stirred overnight at rt. After completion of the reaction according to TLC, the mixture was diluted with water (25 mL) and extracted with EtOAc (3x 20 mL). The combined organic layers were dried over Na_2SO_4, filtered, and concentrated *in vacuo*. The crude methyl 3-(2-(*tert*-butoxy)-2-oxoethoxy)benzoate was dissolved in a mixture of TFA, DCM (30 mL, 1:1) and anisole (100 µL) and stirred at rt for 3 days. The mixture was concentrated *in vacuo* and the resulting solid was crystalized in diisopropyl ether. **19** was obtained as brown solid (2.94 g, 43%). R_f = (EtOAc + acetic acid 2%) = 0.60. ^{1}H-NMR (500 MHz, acetone-*d*$_6$) δ = 7.63 (dt, *J* = 7.6, 1.2 Hz, 1H), 7.54 (dd, *J* = 2.7, 1.5 Hz, 1H), 7.47–7.40 (m, 1H), 7.23 (ddd, *J* = 8.3, 2.7, 1.1 Hz, 1H), 4.81 (s, 2H), 3.88 (s, 3H) ppm. ^{13}C-NMR (126 MHz, acetone-*d*$_6$) δ = 170.0, 166.9, 159.2, 132.5, 130.6, 123.1, 120.4, 115.8, 65.5, 52.4 ppm. (APCI+): *m/z* 211.2 ($[M+H]^+$).

Methyl 3-((6-(3-fluoro-4-methylphenoxy)-1-methyl-1*H*-benzo[*d*]imidazol-2-yl)methoxy)benzoate (20). Synthesis was performed according to General Procedure A using **14d** (250 mg, 1.02 mmol, 1.0 eq.) and 2-(3-(methoxycarbonyl)phenoxy)acetic acid (**19**, 213 mg, 1.02 mmol, 1.0 eq.). **20** was obtained as orange oil (70 mg,

17%). R_f = (cyclohexane/EtOAc 6:4) = 0.61. ^{1}H-NMR (400 MHz, DMSO-d_6): δ = 7.71–7.63 (m, 2H), 7.63–7.57 (m, 1H), 7.53–7.45 (m, 1H), 7.45–7.39 (m, 1H), 7.38–7.33 (m, 1H), 7.29–7.20 (m, 1H), 6.96 (dd, *J* = 8.7, 2.3 Hz, 1H), 6.80 (dd, *J* = 11.2, 2.5 Hz, 1H), 6.76–6.69 (m, 1H), 5.49 (s, 2H), 3.86 (s, 3H), 3.82 (s, 3H), 2.19 (dd, *J* = 2.0, 0.7 Hz, 3H) ppm. ^{13}C-NMR (126 MHz, DMSO-d_6): δ = 166.4, 161.4 (d, *J* = 243.9 Hz), 158.4, 157.8 (d, *J* = 10.6 Hz), 152.4, 150.5, 139.1, 137.1, 132.6 (d, *J* = 6.8 Hz), 131.6, 130.5, 122.6, 120.9, 120.6, 118.7 (d, *J* = 17.3 Hz), 115.6, 115.3, 113.7, 105.4 (d, *J* = 25.3 Hz), 102.1, 63.2, 52.8, 30.6 (d, *J* = 4.0 Hz), 14.0 ppm. (APCI+): *m/z* 421.8 ($[M+H]^+$).

1,5-Difluoro-3-isopropoxy-2-nitrobenzene (22). 3,5-Difluoro-2-nitrophenol (**21**, 2.00 g, 11.2 mmol, 1.0 eq.) was dissolved in dry THF (50 mL). Isopropanol (1.70 mL, 22.3 mmol, 2.0 eq.), PPh_3 (3.25 g, 12.3 mmol, 1.1 eq.) and DBAD (2.88 g, 12.3 mmol, 1.1 eq.) were added at 0 °C. After stirring for 1 h at 0 °C, the solution was allowed to warm to rt and stirred overnight. After removing the solvent *in vacuo* and dissolving the residue in EtOAc (50 mL), the organic phase was washed with 1 N HCl(aq) (2x 50 mL) and sat. NH_4Cl(aq) (1x 50 mL). The organic phase was dried over $MgSO_4$, filtered, and after removing the solvent *in vacuo*, the crude product was purified *via* aFCC (cyclohexane (1% AcOH)) and reverse phase aFCC (water/ACN 7:3 to 0:1) to yield **22** (1.79 g, 74%) as yellow oil. R_f (cyclohexane/EtOAc 8:2) = 0.50. ^{1}H-NMR (400 MHz, $CDCl_3$) δ = 6.59–6.49 (m, 2H), 4.59 (hept, *J* = 6.1 Hz, 1H), 1.38 (d, *J* = 6.0 Hz, 6H) ppm. ^{13}C-NMR (101 MHz, $CDCl_3$) δ = 163.8 (dd, *J* = 253.1, 14.5 Hz), 155.6 (dd, *J* = 257.5, 16.2 Hz), 152.6 (dd, *J* = 13.0, 4.6 Hz), 98.6 (dd, *J* = 26.7, 3.6 Hz), 97.0 (d, *J* = 23.4 Hz), 96.7 (d, *J* = 23.5 Hz), 74.0, 21.7 ppm. (APCI+): *m/z* 217.7 ($[M+H]^+$).

5-Fluoro-3-isopropoxy-*N*-methyl-2-nitroaniline (23). 22 (1.80 g, 8.29 mmol, 1.0 eq.) was dissolved in dry THF (10 mL) and methylamine (2 M in THF; 9.12 mL, 18.2 mmol, 2.2 eq.) was added. The reaction mixture was stirred at 40 °C overnight. After removing the solvent *in vacuo*, EtOAc (50 mL) was added and the mixture was washed with half-saturated brine (2x 50 mL) and brine (1x 50 mL). The organic phase was dried over $MgSO_4$, filtered, and the solvent was removed *in vacuo* to yield **23** (1.88 g, 99%) as orange solid. R_f (cyclohexane/EtOAc 8:2) = 0.72. ^{1}H-NMR (400 MHz, $CDCl_3$) δ = 6.52 (d, *J* = 16.6 Hz, 1H), 6.01 (s, 1H), 5.98 (s, 1H), 4.54 (hept, *J* = 6.1 Hz, 1H), 2.88 (d, *J* = 5.0 Hz, 3H), 1.37 (d, *J* = 6.1 Hz, 6H) ppm. ^{13}C-NMR (101 MHz, $CDCl_3$) δ = 166.2 (d, *J* = 249.6 Hz), 156.1 (d, *J* = 14.6 Hz), 147.1 (d, *J* = 15.1 Hz), 125.1, 90.8 (d, *J* = 27.5 Hz), 90.5 (d, *J* = 27.5 Hz), 72.9, 30.3, 21.9 ppm. (APCI+): *m/z* 228.8 ($[M+H]^+$).

3-Isopropoxy-*N*-methyl-2-nitro-5-(*p*-tolyloxy)aniline (24a). 24a was synthesized according to General Procedure D using **23** (107 mg, 0.469 mmol, 1.0 eq.), *p*-cresol (**12a**, 67.0 mg, 0.620 mmol, 1.3 eq.) and NaH (60% in mineral oil, 35.0 mg, 0.875 mmol, 1.8 eq.) in dry DMF (5 mL). **24a** (97.0 mg, 65%) was obtained as yellow solid. R_f (cyclohexane/EtOAc 8:2) = 0.55. ^{1}H-NMR (400 MHz, $CDCl_3$) δ = 7.22–7.16 (m, 2H), 7.00–6.94 (m, 2H), 6.77 (d, *J* = 5.5 Hz, 1H), 5.86 (dd, *J* = 2.4, 0.6 Hz, 1H), 5.80 (d, *J* = 2.4 Hz, 1H), 4.45 (hept, *J* = 6.2 Hz, 1H), 2.78 (d, *J* = 5.0 Hz, 3H), 2.37 (s, 3H), 1.32 (d, *J* = 6.1 Hz, 6H) ppm. ^{13}C-NMR (101 MHz, $CDCl_3$) δ = 163.0, 156.2, 152.7, 147.5, 134.6, 130.6, 123.7, 120.3, 92.6, 91.9, 72.5, 30.2, 22.0, 21.0 ppm. (APCI+): *m/z* 316.7 ($[M+H]^+$).

5-(3,5-Dimethylphenoxy)-3-isopropoxy-*N*-methyl-2-nitroaniline (24b). 24b was synthesized according to General Procedure D using **23** (203 mg, 0.889 mmol, 1.0 eq.), 3,5-xylenol (**12e**, 133 mg, 1.09 mmol, 1.2 eq.) and NaH (60% in mineral oil, 38.0 mg, 0.950 mmol, 1.1 eq.) in dry DMF (10 mL). **24b** (290 mg, 99%) was obtained as yellow oil. R_f (cyclohexane/EtOAc 8:2) = 0.63. ^{1}H-NMR (400 MHz, $CDCl_3$) δ = 6.83 (tq, *J* = 1.5, 0.7 Hz, 1H), 6.79–6.72 (m, 1H), 6.69 (dq, *J* = 1.4, 0.7 Hz, 2H), 5.87 (dd, *J* = 2.4, 0.6 Hz, 1H), 5.83 (d, *J* = 2.4 Hz, 1H), 4.45 (pd, *J* = 6.1, 0.5 Hz, 1H), 2.80 (d, *J* = 5.0 Hz, 3H), 2.32 (s, 3H), 2.31 (s, 3H), 1.33 (d, *J* = 6.1 Hz, 6H) ppm. ^{13}C-NMR (101 MHz, $CDCl_3$) δ = 162.6, 156.1, 155.2, 147.5, 140.0, 126.4, 123.9, 117.8, 93.1, 92.5, 72.5, 30.2, 21.9, 21.4 ppm. (APCI+): *m/z* 330.8 ($[M+H]^+$).

3-Isopropoxy-*N*-methyl-2-nitro-5-(3-(trifluoromethoxy)phenoxy)aniline (24c). 24c was synthesized according to General Procedure D using **23** (203 mg, 0.889 mmol, 1.0 eq.), 3-(trifluoromethoxy)phenol (**12f**,

125 µL, 0.968 mmol, 1.1 eq.) and NaH (60% in mineral oil, 39.1 mg, 0.978 mmol, 1.1 eq.) in dry DMF (10 mL). **24c** (198 mg, 58%) was obtained as yellow oil. R_f (cyclohexane/EtOAc 8:2) = 0.65. ^{1}H-NMR (400 MHz, $CDCl_3$) δ = 7.40 (t, *J* = 8.3 Hz, 1H), 7.05 (ddt, *J* = 8.3, 2.1, 1.0 Hz, 1H), 7.01 (ddd, *J* = 8.3, 2.3, 0.9 Hz, 1H), 6.96 (tt, *J* = 2.3, 1.1 Hz, 1H), 5.89 (dd, *J* = 2.3, 0.5 Hz, 1H), 5.87 (d, *J* = 2.4 Hz, 1H), 4.47 (hept, *J* = 6.1 Hz, 1H), 2.81 (s, 3H), 1.33 (d, *J* = 6.1 Hz, 6H) ppm. ^{13}C-NMR (101 MHz, $CDCl_3$) δ = 161.2, 156.6, 156.1, 150.2 (q, *J* = 1.9 Hz), 147.3, 130.9, 124.6, 120.5 (q, *J* = 257.7 Hz), 118.0, 116.8, 112.9, 93.1, 93.1, 72.7, 30.2, 21.9 ppm. (APCI+): *m/z* 386.7 ($[M+H]^+$).

3-Isopropoxy-*N*-methyl-2-nitro-5-(3-(trifluoromethyl)phenoxy)aniline (24d). 24d was synthesized according to General Procedure D using **23** (202 mg, 0.885 mmol, 1.0 eq.), 3-(trifluoromethyl)phenol (**12b**, 120 µL, 0.988 mmol, 1.1 eq.) and NaH (60% in mineral oil, 38.0 mg, 0.950 mmol, 1.1 eq.) in dry DMF (10 mL). **24d** (284 mg, 87%) was obtained as yellow oil. R_f (cyclohexane/EtOAc 8:2) = 0.76. ^{1}H-NMR (500 MHz, $CDCl_3$) δ = 7.51 (t, *J* = 8.0 Hz, 1H), 7.44 (dq, *J* = 7.7, 0.9 Hz, 1H), 7.34 (t, *J* = 2.2 Hz, 1H), 7.24 (d, *J* = 2.4 Hz, 1H), 5.88 (d, *J* = 2.4 Hz, 1H), 5.87 (d, *J* = 2.4 Hz, 1H), 4.46 (hept, *J* = 6.2 Hz, 1H), 2.81 (s, 3H), 1.33 (d, *J* = 6.1 Hz, 6H) ppm. ^{13}C-NMR (126 MHz, $CDCl_3$) δ = 167.2, 161.2, 156.1, 156.0, 147.3, 132.6 (q, *J* = 32.9 Hz), 130.7, 123.6 (q, *J* = 250.5 Hz), 123.0, 121.1 (q, *J* = 3.8 Hz), 116.8 (q, *J* = 3.8 Hz), 93.2, 90.6, 72.7, 30.2, 21.9 ppm. (APCI+): *m/z* 370.7 ($[M+H]^+$).

3-Isopropoxy-*N*-methyl-2-nitro-5-(*m*-tolyloxy)aniline (24e). 24e was synthesized according to General Procedure D using **23** (200 mg, 0.876 mmol, 1.0 eq.), *m*-cresol (**12g**, 100 µL, 0.964 mmol, 1.1 eq.) and NaH (60% in mineral oil, 39.0 mg, 0.975 mmol, 1.1 eq.) in dry DMF (10 mL). **24e** (270 mg, 97%) was obtained as yellow oil. R_f (cyclohexane/EtOAc 8:2) = 0.67. ^{1}H-NMR (400 MHz, $CDCl_3$) δ = 7.27 (t, *J* = 7.8 Hz, 1H), 7.01 (ddt, *J* = 7.6, 1.7, 0.8 Hz, 1H), 6.91–6.85 (m, 2H), 5.87 (dd, *J* = 2.4, 0.6 Hz, 1H), 5.83 (d, *J* = 2.4 Hz, 1H), 4.50–4.40 (m, 1H), 2.80 (s, 3H), 2.36 (d, *J* = 0.8 Hz, 3H), 1.32 (d, *J* = 6.1 Hz, 6H) ppm. ^{13}C-NMR (101 MHz, $CDCl_3$) δ = 162.6, 156.2, 155.2, 147.4, 140.4, 129.8, 125.6, 123.9, 120.9, 117.2, 93.0, 92.5, 72.5, 30.2, 21.9, 21.5 ppm. (APCI+): *m/z* 316.7 ($[M+H]^+$).

Methyl 3-(2-((2-isopropoxy-6-(methylamino)-4-(*p*-tolyloxy)phenyl)amino)-2-oxoethoxy)benzoate (26a). 26a was synthesized according to General Procedure F using **24a** (90.0 mg, 0.284 mmol, 1.0 eq.), iron powder (80.0 mg, 1.43 mmol, 5.0 eq.) and NH_4Cl (11.5 mg, 0.215 mmol, 0.76 eq.) in EtOH/water (2:1, 15 mL) for the reduction of the nitro group to obtain **25a**. For the amide coupling, the crude aniline derivative **25a**, **19** (64.9 mg, 0.307 mmol, 1.1 eq.), NMI (80.0 µL, 1.00 mmol, 3.6 eq.) and TCFH (86.0 mg, 0.307 mmol, 1.1 eq.) were used in dry ACN (10 mL). **26a** (65.0 mg, 48%) was obtained over two steps as colorless solid. R_f (cyclohexane/EtOAc 6:4) = 0.50. ^{1}H-NMR (400 MHz, $CDCl_3$) δ = 7.71–7.65 (m, 2H), 7.36 (ddd, *J* = 8.2, 7.6, 0.5 Hz, 1H), 7.28–7.24 (m, 3H), 7.16–7.11 (m, 2H), 6.95–6.90 (m, 2H), 6.49 (s, 2H), 5.38 (s, 2H), 4.83 (hept, *J* = 6.1 Hz, 1H), 3.92 (s, 3H), 3.74 (s, 3H), 2.34 (s, 3H), 1.44 (d, *J* = 6.1 Hz, 6H) ppm. ^{13}C-NMR (101 MHz, $CDCl_3$) δ = 166.9, 158.1, 155.8, 155.2, 150.7, 147.4, 138.1, 132.7, 131.8, 130.3, 129.8, 129.6, 123.0, 119.7, 118.5, 115.8, 99.3, 91.8, 70.9, 63.6, 52.4, 30.7, 22.1, 20.8 ppm. (APCI+): *m/z* 478.9 ($[M+H]^+$).

Methyl 3-(2-((4-(3,5-dimethylphenoxy)-2-isopropoxy-6-(methylamino)phenyl)amino)-2-oxoethoxy)benzoate (26b). 26b was synthesized according to General Procedure F using **24b** (291 mg, 0.881 mmol, 1.0 eq.), iron powder (493 mg, 8.83 mmol, 10 eq.) and NH_4Cl (49.0 mg, 0.916 mmol, 1.0 eq.) in EtOH/water (2:1, 15 mL) for the reduction of the nitro group to obtain **25b**. For the amide coupling, the crude aniline derivative **25b**, **19** (210 mg, 0.994 mmol, 1.1 eq.), NMI (250 µL, 3.14 mmol, 3.6 eq.) and TCFH (270 mg, 0.962 mmol, 1.1 eq.) were used in dry ACN (10 mL). **26b** (334 mg, 77%) was obtained over two steps as colorless solid. R_f (cyclohexane/EtOAc 8:2) = 0.36. ^{1}H-NMR (500 MHz, acetone-*d*$_6$) δ = 8.34 (s, 1H), 7.71–7.66 (m, 2H), 7.50 (t, *J* = 7.9 Hz, 1H), 7.37 (ddd, *J* = 8.4, 2.6, 1.0 Hz, 1H), 6.73 (d, *J* = 2.3 Hz, 1H), 6.64 (d, *J* = 1.6 Hz, 2H), 5.99 (d, *J* = 2.5 Hz, 1H), 5.94 (d, *J* = 2.4 Hz, 1H), 5.03 (d, *J* = 5.3 Hz, 1H), 4.76 (s, 2H), 4.41 (hept, *J* = 6.0 Hz, 1H), 3.89 (s, 3H), 2.70 (d, *J* = 5.2 Hz, 3H), 2.26 (s, 6H), 1.18 (d, *J* = 6.0 Hz, 6H) ppm. ^{13}C-NMR (126 MHz,

acetone-d_6) δ = 167.5, 166.9, 159.0, 158.5, 158.2, 154.9, 148.5, 140.2, 132.7, 130.8, 125.3, 123.4, 120.4, 116.9, 116.5, 108.6, 95.2, 94.7, 71.5, 68.6, 52.5, 30.4, 22.3, 21.3 ppm. (APCI+): *m/z* 492.9 ($[M+H]^+$).

Methyl 3-(2-((2-isopropoxy-6-(methylamino)-4-(3-(trifluoromethoxy)phenoxy)phenyl)amino)-2-oxoethoxy)benzoate (26c). **26c** was synthesized according to General Procedure F using **24c** (195 mg, 0.505 mmol, 1.0 eq.), iron powder (336 mg, 6.02 mmol, 12 eq.) and NH_4Cl (36.0 mg, 0.673 mmol, 1.3 eq.) in EtOH/water (2:1, 6.0 mL) for the reduction of the nitro group to obtain **25c**. For the amide coupling, the crude aniline derivative **25c**, **19** (115 mg, 0.547 mmol, 1.1 eq.), NMI (130 µL, 1.63 mmol, 3.2 eq.) and TCFH (151 mg, 0.538 mmol, 1.1 eq.) were used in dry ACN (5 mL). **26c** (177 mg, 64%) was obtained over two steps as colorless solid. R_f (cyclohexane/EtOAc 6:4) = 0.46. ^{1}H-NMR (400 MHz, $CDCl_3$) δ = 8.41 (s, 1H), 7.78–7.73 (m, 1H), 7.66 (dd, *J* = 2.7, 1.5 Hz, 1H), 7.43 (t, *J* = 8.0 Hz, 1H), 7.33 (t, *J* = 8.3 Hz, 1H), 7.23–7.17 (m, 1H), 6.95 (ddq, *J* = 8.2, 2.3, 1.0 Hz, 2H), 6.89 (dt, *J* = 2.4, 1.2 Hz, 1H), 6.33 (s, 1H), 6.16 (d, *J* = 2.4 Hz, 1H), 4.76 (s, 2H), 4.41 (hept, *J* = 6.0 Hz, 1H), 3.93 (s, 3H), 2.83 (s, 3H), 1.23 (d, *J* = 6.1 Hz, 6H) ppm. ^{13}C-NMR (126 MHz, $CDCl_3$) δ = 167.4, 166.6, 157.3, 156.6, 155.0, 150.4, 147.2 (q, *J* = 5.8 Hz), 132.0, 130.9, 130.3, 123.7, 120.5 (q, *J* = 257.6 Hz), 119.3, 117.2, 116.8, 116.4, 115.6, 112.2, 111.7, 105.1, 92.5, 72.1, 67.9, 52.5, 31.7, 21.9 ppm. (APCI+): *m/z* 548.9 ($[M+H]^+$).

Methyl 3-(2-((2-isopropoxy-6-(methylamino)-4-(3-(trifluoromethyl)phenoxy)phenyl)amino)-2-oxoethoxy)benzoate (26d). **26d** was synthesized according to General Procedure F using **24d** (279 mg, 0.753 mmol, 1.0 eq.), iron powder (445 mg, 7.97 mmol, 11 eq.) and NH_4Cl (41.0 mg, 0.766 mmol, 1.0 eq.) in EtOH/water (2:1, 15 mL) for the reduction of the nitro group to obtain **25d**. For the amide coupling, the crude aniline derivative **25d**, **19** (175 mg, 0.833 mmol, 1.1 eq.), NMI (220 µL, 2.76 mmol, 3.6 eq.) and TCFH (230 mg, 0.820 mmol, 1.0 eq.) were used in dry ACN (10 mL). **26d** (157 mg, 39%) was obtained over two steps as colorless solid. R_f (cyclohexane/EtOAc 6:4) = 0.43. ^{1}H-NMR (500 MHz, acetone-d_6) δ = 8.39 (s, 1H), 7.72–7.66 (m, 2H), 7.62–7.57 (m, 1H), 7.51 (t, *J* = 7.9 Hz, 1H), 7.44–7.40 (m, 1H), 7.38 (ddd, *J* = 8.2, 2.7, 1.0 Hz, 1H), 7.32–7.26 (m, 2H), 6.10 (d, *J* = 2.4 Hz, 1H), 6.03 (d, *J* = 2.4 Hz, 1H), 5.13 (d, *J* = 5.3 Hz, 1H), 4.77 (s, 2H), 4.46 (hept, *J* = 6.0 Hz, 1H), 3.90 (s, 3H), 2.73 (d, *J* = 5.2 Hz, 3H), 1.19 (d, *J* = 6.0 Hz, 6H) ppm. ^{13}C-NMR (126 MHz, acetone-d_6) δ = 167.6, 166.9, 159.5, 159.0, 156.9, 155.3, 148.9, 132.7, 132.3 (q, *J* = 32.0 Hz), 131.8, 130.8, 125.0 (q, *J* = 271.7 Hz), 123.4, 122.3, 120.4, 119.9 (q, *J* = 4.0 Hz), 116.5, 115.0 (q, *J* = 3.8 Hz), 109.5, 95.7, 95.1, 71.6, 68.6, 52.5, 30.4, 22.3 ppm. (APCI+): *m/z* 532.9 ($[M+H]^+$).

Methyl 3-(2-((2-isopropoxy-6-(methylamino)-4-(*m*-tolyloxy)phenyl)amino)-2-oxoethoxy)benzoate (26e). **26e** was synthesized according to General Procedure F using **24e** (265 mg, 0.838 mmol, 1.0 eq.), iron powder (664 mg, 11.9 mmol, 14 eq.) and NH_4Cl (44.0 mg, 0.823 mmol, 1.0 eq.) in EtOH/water (2:1, 15 mL) for the reduction of the nitro group to obtain **25e**. For the amide coupling, the crude aniline derivative **25e**, **19** (198 mg, 0.691 mmol, 0.82 eq.), NMI (190 µL, 2.38 mmol, 2.8 eq.) and TCFH (221 mg, 0.788 mmol, 0.94 eq.) were used in dry ACN (8.0 mL). **26e** (288 mg, 72%) was obtained over two steps as colorless solid. R_f (cyclohexane/EtOAc 8:2) = 0.38. ^{1}H-NMR (400 MHz, $CDCl_3$) δ = 8.46 (s, 1H), 7.75 (dt, *J* = 7.9, 1.2 Hz, 1H), 7.66 (dd, *J* = 2.7, 1.5 Hz, 1H), 7.43 (t, *J* = 8.0 Hz, 1H), 7.24–7.18 (m, 2H), 6.95–6.90 (m, 1H), 6.86–6.80 (m, 2H), 6.37 (s, 1H), 6.19 (d, *J* = 2.4 Hz, 1H), 4.76 (s, 2H), 4.40 (hept, *J* = 6.1 Hz, 1H), 3.93 (s, 3H), 2.83 (s, 3H), 2.34 (d, *J* = 0.7 Hz, 3H), 1.22 (d, *J* = 6.0 Hz, 6H) ppm. ^{13}C-NMR (101 MHz, $CDCl_3$) δ = 167.1, 157.4, 157.1, 152.4, 140.5, 140.1, 132.1, 132.0, 130.1, 129.9, 129.6, 124.3, 123.8, 119.8, 119.4, 115.8, 115.6, 101.4, 97.9, 95.6, 71.5, 68.0, 52.5, 31.6, 22.1, 21.6 ppm. (APCI+): *m/z* 478.9 ($[M+H]^+$).

***N*-(*tert*-Butyl)-1-phenylmethanesulfonamide (32a)**. Diisopropylethylamine (3.19 g, 31.5 mmol, 3.0 eq.) was added to a solution of benzylsulfonyl chloride (**30**, 2.00 g, 10.5 mmol, 1.0 eq.) and *tert*-butylamine (**31a**, 3.07 g, 42.0 mmol, 4.0 eq.) in CH_2Cl_2 (40 mL) at 0 °C. The reaction mixture was allowed to warm to rt and stirred overnight. After 21 h the solvent was removed *in vacuo*. The residue was dissolved in EtOAc and washed with sat. aqueous NH_4Cl (2 x 50 mL), sat. aqueous $NaHCO_3$ (2 x 50 mL) and brine (50 mL). The organic layer was dried over $MgSO_4$, filtered, and concentrated *in vacuo*. The crude product was purified by aFCC

(cyclohexane/EtOAc: 98/2 - 85/15) to yield **26a** (1.49 g, 63%) as colorless solid. R_f = (cyclohexane/EtOAc 9:1) = 0.16. ^{1}H-NMR (400 MHz, $CDCl_3$): δ = 7.42–7.34 (m, 5H), 4.24 (s, 2H), 3.92 (s, 1H), 1.35 (s, 9H) ppm. ^{13}C-NMR (101 MHz, $CDCl_3$): δ = 130.9, 130.1, 128.8, 128.7, 62.0, 55.1, 30.4 ppm. (APCI+): *m/z* 227.7 ($[M+H]^+$).

***N*-Isobutyl-1-phenylmethanesulfonamide (32b).** Benzylsulfonyl chloride (**30**, 3.00 g, 15.7 mmol, 1.0 eq.) was added to a solution of isobutylamine (**31b**, 1.73 g, 23.6 mmol, 1.5 eq.) and TEA (3.29 mL, 23.6 mmol, 1.5 eq.) in CH_2Cl_2 (40 mL) at 0 °C. After stirring the reaction mixture for 2 h at rt, the solvent was removed *in vacuo*. The crude product was purified by aFCC (cyclohexane/EtOAc: 98/2 - 85/15) to yield **32b** (567 mg, 16%) as colorless solid. R_f = (cyclohexane/EtOAc 9:1) = 0.15. ^{1}H-NMR (400 MHz, $CDCl_3$): δ = 7.43–7.34 (m, 5H), 4.25 (s, 2H), 4.12 (t, *J* = 6.3 Hz, 1H), 2.81 (t, *J* = 6.5 Hz, 2H), 1.71 (dhept, *J* = 13.4, 6.7 Hz, 1H), 0.89 (d, *J* = 6.7 Hz, 6H) ppm. ^{13}C-NMR (101 MHz, $CDCl_3$): δ = 130.7, 129.6, 129.0, 128.9, 58.7, 51.2, 29.2, 19.9 ppm. (APCI+): *m/z* 227.7 ($[M+H]^+$).

***N*-(4-Bromobenzyl)-*N*-(*tert*-butyl)-1-phenylmethanesulfonamide (34a).** Synthesis of **34a** was performed according to General Procedure G using **32a** (200 mg, 0.880 mmol, 1.0 eq.), NaH (60% in mineral oil, 25.4 mg, 0.636 mmol, 0.72 eq.) and 4-bromobenzyl bromide (**33**, 242 mg, 0.968 mmol, 1.2 eq.) in *N*,*N*-dimetylacetamide (3.0 mL). The reaction mixture was stirred for 22 h. **34a** (254 mg, 29%) was obtained as colorless solid. R_f = (cyclohexane/EtOAc 8:2) = 0.63. ^{1}H-NMR (400 MHz, $CDCl_3$): δ = 7.45–7.33 (m, 7H), 7.24–7.15 (m, 2H), 4.31 (s, 2H), 4.01 (s, 2H), 1.42 (s, 9H) ppm. ^{13}C-NMR (101 MHz, $CDCl_3$): δ = 138.6, 131.5, 130.9, 129.7, 129.6, 128.9, 128.8, 121.1, 62.0, 60.2, 50.5, 30.8 ppm. (APCI+): *m/z* 397.6 ($[M+H]^+$).

***N*-(4-Bromobenzyl)-*N*-isobutyl-1-phenylmethanesulfonamide (34b).** Synthesis of **34b** was performed according to General Procedure G using **32b** (150 mg, 0.660 mmol, 1.0 eq.), NaH (60% in mineral oil, 19.1 mg, 0.476 mmol, 0.72 eq.) and 4-bromobenzyl bromide (**33**, 181 mg, 0.726 mmol, 1.2 eq.) in *N*,*N*-dimetylacetamide (3.0 mL). The reaction mixture was stirred for 16 h. **34b** (191 mg, 73%) was obtained as colorless solid. R_f = (cyclohexane/EtOAc 9:1) = 0.38. ^{1}H-NMR (400 MHz, $CDCl_3$): δ = 7.50–7.40 (m, 2H), 7.40–7.30 (m, 5H), 7.23–7.15 (m, 2H), 4.21 (s, 2H), 4.07 (s, 2H), 2.83 (d, *J* = 7.5 Hz, 2H), 1.56 (m, 1H), 0.75 (d, *J* = 6.7 Hz, 6H) ppm. ^{13}C-NMR (101 MHz, $CDCl_3$): δ = 136.2, 131.7, 130.8, 130.3, 129.1, 128.90, 128.86, 121.9, 58.2, 57.2, 27.1, 20.0 ppm. (APCI+): *m/z* 397.6 ($[M+H]^+$).

***N*-*tert*-Butyl-4-iodobenzene-1-sulfonamide (37a).** 4-Iodbenzenesulfonyl chloride (**30**, 1.00 g, 3.08 mmol, 1.0 eq.) was dissolved in CH_2Cl_2 (0.2 M) and added dropwise to a separate flask filled with *tert*-butylamine (**31a**, 482 µL, 4.61 mmol, 1.5 eq.) and TEA (1.07 mL, 7.69 mmol, 2.5 eq.) in CH_2Cl_2 (0.2 M). The reaction was stirred at rt overnight. After completion according to TLC, the reaction mixture was concentrated *in vacuo*. The crude was diluted with water (25 mL) and extracted with EtOAc (3x 20 mL). The combined organic layers were dried over Na_2SO_4, filtered, and concentrated *in vacuo.* Further purification was performed by aFCC (cyclohexane/EtOAc 8:2). **37a** was obtained as a transparent solid (729 mg, 70%). R_f = (cyclohexane/EtOAc 8:2) = 0.20. ^{1}H-NMR (400 MHz, $CDCl_3$): δ = 7.81–7.73 (m, 2H), 7.59–7.51 (m, 2H), 4.98 (s, 1H), 1.15 (s, 9H) ppm. ^{13}C-NMR (101 MHz, $CDCl_3$): δ = 143.4, 138.2, 128.5, 99.4, 55.0, 30.2 ppm. (APCI+): *m/z* 340.1 ($[M+H]^+$).

4-Iodo-*N*-(2-methylpropyl)benzene-1-sulfonamide (37b). 4-Iodbenzenesulfonyl chloride (**36**, 1.00 g, 3.08 mmol, 1.0 eq.) was dissolved in CH_2Cl_2 (0.2 M) and added dropwise to a separate flask filled with isobutylamine (**31b**, 458 µL, 4.61 mmol, 1.5 eq.) and TEA (1.07 mL, 7.69 mmol, 2.5 eq.) in CH_2Cl_2 (0.2 M). The reaction was stirred at rt overnight. After completion according to TLC, the reaction mixture was concentrated *in vacuo*. The crude was diluted with water (25 mL) and extracted with EtOAc (3x 20 mL). The combined organic layers were dried over Na_2SO_4, filtered, and concentrated *in vacuo.* Further purification was performed by aFCC (cyclohexane/EtOAc 8:2). **37b** was obtained as a transparent solid (495 mg, 47%). R_f = (cyclohexane/EtOAc 8:2) = 0.45. ^{1}H-NMR (400 MHz, $CDCl_3$): δ = 7.91–7.83 (m, 2H), 7.61–7.53 (m, 2H), 4.41 (t, *J* = 6.4 Hz, 1H), 2.77 (t, *J* = 6.6 Hz, 2H), 1.79–1.65 (m, 1H), 0.88 (d, *J* = 6.7 Hz, 6H) ppm. ^{13}C-NMR (101 MHz, $CDCl_3$): δ = 139.9, 138.4, 128.5, 99.9, 50.6, 28.5, 19.8 ppm. (APCI+): *m/z* 340.1 ($[M+H]^+$).

***N*-*tert*-Butyl-4-iodo-*N*-((2-(trifluoromethyl)phenyl)methyl)benzene-1-sulfonamide (39a).** To a stirred solution of *N*-tert-butyl-4-iodobenzene-1-sulfonamide (**37a**, 487 mg, 1.44 mmol, 1.0 eq.) and NaH (60% in mineral oil, 115 mg, 2.87 mmol, 2.0 eq.) in DMSO (0.1 M) was added 2-trifluoromethylbenzyl bromide (**38**, 451 µL, 2.87 mmol, 2.0 eq.). The reaction was stirred at rt overnight. After completion according to TLC, the reaction mixture was diluted with water (25 mL) and extracted with EtOAc (3x 20 mL). The combined organic layers were dried over Na_2SO_4, filtered, and concentrated *in vacuo.* Further purification was performed by reverse phase aFCC (water/ACN 8:2 to ACN 100%). **39a** was obtained as transparent solid (490 mg, 67%). R_f = (cyclohexane/EtOAc 8:2) = 0.70. ^{1}H-NMR (400 MHz, $CDCl_3$): δ = 7.86–7.76 (m, 3H), 7.58–7.47 (m, 4H), 7.33–7.24 (m, 1H), 4.78 (s, 2H), 1.20 (s, 9H) ppm. ^{13}C-NMR (101 MHz, $CDCl_3$): δ = 142.6, 138.5 (d, *J* = 1.5 Hz), 138.3 132.1, 128.8, 128.7, 128.7-124.3 (q, *J* = 270 Hz), 126.9, 126.6-125.6 (q, *J* = 31 Hz), 125.7 (q, *J* = 5.9 Hz), 99.6, 60.5, 47.4 (q, *J* = 3.4 Hz), 30.0 ppm. (APCI+): *m/z* 497.1 ($[M+H]^+$).

4-Iodo-*N*-(2-methylpropyl)-*N*-((2-(trifluoromethyl)phenyl)methyl)benzene-1-sulfonamide (39b). To a stirred solution of 4-iodo-*N*-(2-methylpropyl)benzene-1-sulfonamide (**37b**, 446 mg, 1.32 mmol, 1.0 eq.) and NaH (60% in mineral oil, 105 mg, 2.63 mmol, 2.0 eq.) in DMSO (0.1 M) was added 2-trifluoromethylbenzyl bromide (**38**, 410 µL, 2.63 mmol, 2.0 eq.). The reaction was stirred at rt overnight. After completion according to TLC, the reaction mixture was diluted with water (25 mL) and extracted with EtOAc (3x 20 mL). The combined organic layers were dried over Na_2SO_4, filtered, and concentrated *in vacuo.* Further purification was performed by reverse phase aFCC (water/ACN 8:2 to ACN 100%). **39b** was obtained as transparent oil (596 mg, 91%). R_f = (cyclohexane/EtOAc 8:2) = 0.65. ^{1}H-NMR (400 MHz, $CDCl_3$): δ = 7.94–7.87 (m, 2H), 7.84 (d, *J* = 7.9 Hz, 1H), 7.64–7.53 (m, 4H), 7.38 (t, *J* = 7.7 Hz, 1H), 4.48 (s, 2H), 2.95 (d, *J* = 7.4 Hz, 2H), 1.56–1.41 (m, 1H), 0.73 (d, *J* = 6.7 Hz, 6H) ppm. ^{13}C-NMR (101 MHz, $CDCl_3$): δ = 139.2, 138.6, 136.1–135.8 (m), 132.3, 129.9, 128.7, 127.6, 127.4 (q, *J* = 30.5 Hz), 125.8 (q, *J* = 6.0 Hz), 128.5-123.0 (q, *J* = 270 Hz), 100.2, 58.2, 49.7 (d, *J* = 2.9 Hz), 27.0, 20.1 ppm. (APCI+): *m/z* 497.1 ($[M+H]^+$).

Reporter gene assays

Modulation of PPARγ and RORγ by the CLM designed compounds was tested in Gal4 hybrid reporter gene assays using the Gal4-fusion receptor plasmids pFA-CMV-hPPARγ-LBD[11] and pFA-CMV-hRORγ-LBD[12], coding for the hinge region and ligand binding domain of the canonical isoform of the respective human nuclear receptor, the reporter plasmid pFR-Luc (StrateGene, La Jolla, California, USA), and pRL-SV40 (Promega, Madison, Wisconsin, USA) constitutively expressing Renilla luciferase for normalization of transfection efficiency and test compound toxicity. HEK293T cells (American Type Culture Collection (ATCC); CRL-3216) were cultured in Dulbecco's modified Eagle's medium (DMEM), high glucose supplemented with 10% fetal calf serum (FCS), sodium pyruvate (1 mM), penicillin (100 U/mL), and streptomycin (100 µg/mL) at 37 °C and 5% CO2, and seeded in 96-well plates (3 x 104 cells/well) on the day before transfection. After 24 h, the medium was changed to Opti-MEM without supplements, and the cells were transiently transfected with one Gal4-fusion receptor plasmid, pFR-Luc and pRL-SV40 using Lipofectamine LTX reagent (Invitrogen, Carlsbad, California, USA) according to the manufacturer's protocol. Five hours after transfection, cells were incubated with the test compounds in Opti-MEM supplemented with penicillin (100 U/mL), streptomycin (100 µg/mL), and 0.1% DMSO for 16 h before luciferase activity was measured using the Dual-Glo Luciferase Assay System (Promega) according to the manufacturer's protocol on a Tecan Spark Cyto (Tecan Group AG, Männedorf, Switzerland) plate reader. Firefly luminescence was divided by Renilla luminescence and multiplied by 1000 resulting in relative light units (RLU) to normalize for transfection efficiency and cell growth. Fold activation was obtained by dividing the mean RLU of a test sample by the mean RLU of the untreated control (0.1% DMSO). Pioglitazone (1 µM, PPARγ) and T0901317 (1 µM, RORγ) were used as positive controls to monitor assay performance. All samples were tested in at least three biologically independent experiments in duplicates. For dose-response curve fitting, the fold activation values were used with the equation "[Agonist] vs. response (three parameters)" in GraphPad Prism 7 (GraphPad Software, La Jolla, CA, USA).

Supplementary References

1. Shinozuka, T. *et al.* Discovery of DS-6930, a potent selective PPARγ modulator. Part II: Lead optimization. *Bioorganic Med. Chem.* **26**, 5099–5117 (2018).
2. Yanagisawa, H. *et al.* Novel oximes having 5-benzyl-2,4-thiazolidinedione as antihyperglycemic agents: Synthesis and structure-activity relationship. *Bioorganic Med. Chem. Lett.* **10**, 373–375 (2000).
3. Sonawane, L. V. & Bari, S. B. Ligand-based in silico 3D-QSAR study of PPAR-c agonists. *Med. Chem. Res.* **20**, 1005–1014 (2011).
4. Shinozuka, T. *et al.* Structure-Activity Relationship Studies of 3- or 4-Pyridine Derivatives of DS-6930. *ACS Med. Chem. Lett.* **10**, 358–362 (2019).
5. Shinozuka, T. *et al.* Discovery of DS-6930, a potent selective PPARγ modulator. Part I: Lead identification. *Bioorganic Med. Chem.* **26**, 5079–5098 (2018).
6. Sun, N. *et al.* Discovery of novel N-sulfonamide-tetrahydroquinolines as potent retinoic acid receptor-related orphan receptor γt inverse agonists for the treatment of autoimmune diseases. *Eur. J. Med. Chem.* **187**, 111984 (2020).
7. Fauber, B. P. *et al.* Reduction in lipophilicity improved the solubility, plasma-protein binding, and permeability of tertiary sulfonamide RORc inverse agonists. *Bioorganic Med. Chem. Lett.* **24**, 3891–3897 (2014).
8. Van Niel, M. B. *et al.* A reversed sulfonamide series of selective RORc inverse agonists. *Bioorganic Med. Chem. Lett.* **24**, 5769–5776 (2014).
9. Fauber, B. P. *et al.* Identification of tertiary sulfonamides as RORc inverse agonists. *Bioorganic Med. Chem. Lett.* **24**, 2182–2187 (2014).
10. Pauli, G. F. *et al.* Importance of purity evaluation and the potential of quantitative 1H NMR as a purity assay. *J. Med. Chem.* **57**, 9220–9231 (2014).
11. Arifi, S. *et al.* Targeting the Alternative Vitamin E Metabolite Binding Site Enables Noncanonical PPARγ Modulation. *J. Am. Chem. Soc.* **145**, 14802–14810 (2023).
12. Moret, M., Helmstädter, M., Grisoni, F., Schneider, G. & Merk, D. Beam Search for Automated Design and Scoring of Novel ROR Ligands with Machine Intelligence**. *Angew. Chemie - Int. Ed.* **60**, 19477–19482 (2021).
