# Supplementary material for: Structural optimization of drug molecules with incrementally trained language models
Source: Nat Commun. 2026 Apr 11;17:3456. doi: 10.1038/s41467-026-71591-w (PMC13076696; doi:10.1038/s41467-026-71591-w)
Supplement: Supplementary file 2 — Description of Additional Supplementary Files [file 41467_2026_71591_MOESM2_ESM.pdf]

Description of Additional Supplementary Files

Supplementary Data 1

Description: contains full lists of CLM designs obtained from different training strategies/ 5 models, pretraining data, results of retrospective evaluation, and PPARγ and RORγ ligand SAR series 6 used for CLM fine-tuning.
